# Exosome-Mediated Differentiation of Mouse Embryonic Fibroblasts and Exocrine Cells into β-Like Cells and the Identification of Key miRNAs for Differentiation

**DOI:** 10.3390/biomedicines8110485

**Published:** 2020-11-09

**Authors:** Paulami Mandal, Debojyoti De, Dong Uk Im, Sung Hee Um, Kyeong Kyu Kim

**Affiliations:** 1 Department of Precision Medicine, Sungkyunkwan University School of Medicine, Suwon 16419, Korea; mandalpaulami28@gmail.com; 2 Department of Biotechnology, National Institute of Technology, Durgapur 713209, India; debojyoti.de@bt.nitdgp.ac.in; 3 Department of Molecular Cell Biology, Sungkyunkwan University School of Medicine, Suwon 16419, Korea; Ldw4545@gmail.com (D.U.I.); shum@skku.edu (S.H.U.)

**Keywords:** diabetes, exosomes, differentiation, miRNA, small molecules, β-cell, exocrine cells

## Abstract

Diabetes is a concerning health malady worldwide. Islet or pancreas transplantation is the only long-term treatment available; however, the scarcity of transplantable tissues hampers this approach. Therefore, new cell sources and differentiation approaches are required. Apart from the genetic- and small molecule-based approaches, exosomes could induce cellular differentiation by means of their cargo, including miRNA. We developed a chemical-based protocol to differentiate mouse embryonic fibroblasts (MEFs) into β-like cells and employed mouse insulinoma (MIN6)-derived exosomes in the presence or absence of specific small molecules to encourage their differentiation into β-like cells. The differentiated β-like cells were functional and expressed pancreatic genes such as Pdx1, Nkx6.1, and insulin 1 and 2. We found that the exosome plus small molecule combination differentiated the MEFs most efficiently. Using miRNA-sequencing, we identified miR-127 and miR-709, and found that individually and in combination, the miRNAs differentiated MEFs into β-like cells similar to the exosome treatment. We also confirmed that exocrine cells can be differentiated into β-like cells by exosomes and the exosome-identified miRNAs. A new differentiation approach based on the use of exosome-identified miRNAs could help people afflicted with diabetes

## 1. Introduction

Diabetes mellitus is a chronic metabolic condition that arises due to the inability of the body to produce or respond efficiently to insulin. There are two major forms of diabetes—type 1 diabetes (T1D) and type 2 diabetes (T2D) [1]. T1D occurs when the body produces autoantibodies that destroy pancreatic β-cells. T2D results from increased insulin resistance in peripheral tissues such as muscle and adipose tissue [2]. For the long-term treatment of diabetes, pancreas or islet cell transplantation is required. However, the demand for islet cells far exceeds the supply. Therefore, alternative strategies for the treatment of diabetes, including cell therapy, are being investigated [3]. Cell therapy encompasses the differentiation of stem cells ((human embryonic stem cells (ESCs), human-induced pluripotent stem cells (iPSCs) and adult stem cells like mesenchymal stem cells (MSCs)) and direct differentiation of somatic cells using small molecules, the latter process is favored due to its clinical safety. During the differentiation of stem cells and somatic cells such as fibroblasts into pancreatic β-like cells, the differentiating cells often retrace the path of pancreatic organogenesis—definitive endoderm, pancreatic endoderm, pancreatic progenitors, and finally pancreatic islet generation [4,5,6].

Recently, a new mode of intercellular communication mediated by extracellular-vesicles (EVs) was discovered. EVs include apoptotic bodies (500–1000 nm), microvesicles (100 or 200–1000 nm), or exosomes (30–200 nm), depending on their mode of biogenesis [7]. The exosomes contain endosome-related proteins such as Alix, and TSG101, and tetraspanins such as CD63, CD9, CD81, CD82, and CD151 [7,8]. Exosomes are rich in proteins, DNA (genomic and mitochondrial) and RNA, especially small non-coding RNA, including microRNA (miRNA), small nuclear RNA, vault RNA, and Y RNA. The RNA cargo of the exosomes represents a heavily biased subpopulation of the cellular RNAs due to the high selectivity imposed during exosomal cargo loading [9]. To facilitate intercellular communication, exosomes transfer cargo to recipient cells by means of fusion with the plasma- or endosome-membrane or internalization by the cellular endocytic pathways [10]. This mode of communication is highly specific [11], and hence, exosome-mediated transfer of bioactive molecules from donor cells to recipient cells can be used to facilitate cellular differentiation. For example, when ultrasound-stimulated human dermal fibroblasts (HDFs) are grown in neural stem cell medium, they release pro-neural exosomes that carry reprogramming factors such as MYC, SOX2, KLF4, and TCF3 genes, chromatin remodeling proteins, and neurogenesis-related miRNAs that rapidly and efficiently convert the HDFs into neural-progenitor cells [12]. Several other reports have also revealed that exosome-mediated cellular differentiation is possible [13,14,15].

Inspired by those reports, we investigated the role played by insulinoma-derived exosomes in differentiating somatic mouse embryonic fibroblasts (MEFs) into β-like cells. We hypothesized that lineage-specific signals carried by the exosomes would turn on the pancreatic transcriptional program in the MEFs. For this purpose, we exposed the MEFs to mouse insulinoma (MIN6)-derived exosomes and observed that the exosomes differentiated the MEFs into functional β-like cells, and the addition of small molecules augmented the differentiation potential. Moreover, the exosomes differentiated mouse exocrine cells into β-like cells. We found that exosome-identified miRNAs, mmu-miR-709 (miR-709) and mmu-miR-127-5p (miR-127), and their combination successfully prompted MEFs and exocrine cells to undergo β-like cell differentiation. To our knowledge, this is the first study to elucidate the differentiation of MEFs and exocrine cells into β-like cells using exosomes, with small molecules augmenting the differentiation efficiency, and to identify the key miRNAs that enable the exosome-mediated differentiation process.

## 2. Experimental Section

### 2.1. Cell Culture

The INS-1 (rat) and MIN6 (mouse) insulinoma cell lines were a kind gift from Professor Um Sung Hee, Sungkyunkwan University, Republic of Korea. The MIN6 cells were maintained in Dulbecco’s modified Eagle medium (DMEM) (Welgene, Inc., Gyeongsan, Korea) supplemented with exosome-depleted 15% fetal bovine serum (FBS, Gibco, Thermo Fisher Scientific, Inc., Waltham, MA, USA), 1× penicillin-streptomycin (P/S, Welgene, Inc., Gyeongsan, Korea), and 55 µM freshly added β-mercaptoethanol (β-ME) (Sigma Aldrich, Inc., Saint Louis, MO, USA). The INS-1 cells were grown in Roswell Park Memorial Institute Medium (RPMI) 1640 (Welgene, Inc., Gyeongsan, Korea) supplemented with 10% exosome-depleted FBS, 1 mM sodium pyruvate (Gibco, Thermo Fisher Scientific, Inc., Waltham, MA, USA), and 50 µM β-ME (Sigma Aldrich, Inc., Saint Louis, MO, USA) in the presence of 1× P/S. The mouse acinar cell line, 266-6 (ATCC, Manassas, VA, USA), was grown in gelatin-coated tissue culture plates in DMEM, 10% FBS, and 1× P/S, according to the ATCC protocol. All cells were grown in an atmosphere of 5% CO_2_ at 37 °C.

### 2.2. Exosome Isolation and Transmission Electron Microscopy (TEM)

For exosome isolation, 350 mL of MIN6-derived conditioned medium (CM) was collected from 8 T75 tissue culture-treated flasks (Nunc, Sigma Aldrich, Inc., Saint Louis, MO, USA) (1.5 × 10^7^ cells/T75 flask). The CM was differentially centrifuged −750× *g*, 10 min at room temperature (RT) followed by 2000× *g*, 20 min at 4 °C. Next, the supernatant was passed through a 0.22 µm bottle top filter (Corning, Sigma Aldrich, Inc., Saint Louis, MO, USA) to remove contaminating apoptotic bodies, microvesicles, and remaining smaller cell debris and then concentrated using an Amicon^®^ stirred cell (Merck KGaA, Darmstadt, Germany) with 100 kDa ultrafiltration discs (Merck KGaA, Darmstadt, Germany) to a final volume of 50 mL, which was centrifuged using a Beckman Coulter Optima™ XL-100K Ultracentrifuge at 118,000× *g* (40,000 rpm) with a 70Ti rotor (k-factor: 133.7) for 90 min at 4 °C to pellet the exosomes. The supernatant was discarded, and the pellet (in phosphate buffered saline, PBS) was centrifuged again for 90 min at 118,000× *g*. The final pellet was resuspended in 350 µL 1× PBS. The exosomes from INS-1 cells were isolated using the same procedure.

For TEM imaging, the exosome suspension was mixed with an equal volume of 4% paraformaldehyde (Sigma Aldrich, Inc., Saint Louis, MO, USA), deposited on Formvar carbon-coated grids (Electron Microscopy Science, Hatfield, PA, USA), washed with 1× PBS once, and deposited with a 50-µL drop of 1% glutaraldehyde (Sigma Aldrich, Inc., Saint Louis, MO, USA). The samples were washed with water several times, contrasted and embedded in a mixture of 4% uranyl acetate (Sigma Aldrich, Inc., Saint Louis, MO, USA) and 2% methylcellulose (Sigma Aldrich, Inc., Saint Louis, MO, USA) as described previously [16], and viewed under the TEM (JEM ARM 200F, JEOL Ltd., Japan).

### 2.3. Labeling of the MIN6-Derived Exosomes

The MIN6-derived exosomes were labeled using an ExoGlow EV membrane labeling kit (System Biosciences, Palo Alto, CA, USA) according to the manufacturer’s instructions. Briefly, 12 µL of reaction buffer was added to 2 µL of labeling dye (red) and mixed. Exosome solution (dissolved in 1× PBS) equivalent to 50 µg of protein (BCA assay, Pierce™ BCA Protein Assay Kit, Thermo Fisher Scientific, Inc., Waltham, MA, USA) was added to the reaction mixture and incubated at RT for 30 min. To remove free dye molecules, 35 μL of ExoQuick-TC (System Biosciences, Palo Alto, CA, USA) was added to the exosome mixture and kept for 30 min at 4 °C to precipitate the labeled exosomes. The mixture was centrifuged at 10,000 rpm for 10 min at room temperature (RT). The supernatant containing the free dye molecules was carefully aspirated and discarded. The labeled exosome pellet was resuspended in 1× PBS. As a control, we used an equal volume of 1× PBS for dye labeling because the exosomes were resuspended in PBS after their isolation.

### 2.4. Internalization of ExoGlow-Labeled MIN6 Exosomes by MEFs

First, 50 µg of labeled exosomes or an equal volume of labeled 1× PBS was mixed with 1 mL of complete DMEM and incubated with MEFs seeded at a density of 1 × 10^5^ cells/well in 12-well plates (Techno Plastic Products AG, Trasadingen, Switzerland) for 72 h at 37 °C for cellular internalization. Then, we used live cell imaging with a fluorescence microscope (Olympus IX710, Tokyo, Japan) to visualize the internalized labeled exosomes.

For concentration-dependent internalization experiments 10 µg, 50 µg, and 100 µg of MIN6-derived exosomes were labeled with ExoGlow dye as mentioned previously. For the labeling of PBS, a volume equivalent to 100 µg exosomes was taken and ExoGlow labeling was done. These exosomes were then resuspended in 1 mL of complete DMEM and added to MEFs seeded in tissue culture plates and incubated for 72 h at 37 °C.

For measuring the mean fluorescence intensity, ImageJ (NIH, Bethesda, MD, USA) software (version 1.53c) was used and an arbitrary unit (a.u.) was used as the fluorescence unit. Intensities of the fluorescent cells and the background was measured using the free selection tool available in ImageJ and mean fluorescence intensity was calculated with slight modifications as described previously [17].

### 2.5. Determination of the Total Protein Concentration of the Exosomes

A small aliquot of the exosomes was disrupted in 1× radio-immunoprecipitation assay (RIPA) lysis buffer (Cell Signaling Technology, Inc., Danvers, MA, USA) in the presence of phenylmethylsulfonyl fluoride (Sigma Aldrich, Inc., Saint Louis, MO, USA). The mixture was then sonicated thrice for 5 min with intermittent vortexing. The total protein concentration was quantified using a bicinchoninic acid (BCA) protein assay according to the manufacturer’s protocol.

### 2.6. Quantitation of the Number of Exosomes

The exosomes were quantitated using an EXOCET Exosome Quantitation Assay (System Biosciences, Palo Alto, CA, USA) according to the manufacturer’s instructions. This assay is based on the activity of the acetyl-CoA acetylcholinesterase known to be enriched in exosomes. Briefly, the exosome volume corresponding to 50 µg of protein (BCA assay) was determined. That volume was lysed with lysis buffer and centrifuged at 1500× *g* for 5 min, and then the supernatant was collected. 50 µL of the reaction buffer (combining supplied buffers A and B) and 50 µL of the exosome protein (total volume = 100 µL) was added to each well of the microtiter plate, incubated for 20 min at RT, and read at 405 nm. The assay determined the number of exosomes; 50 µg of exosome protein yielded an average of 3–5 × 10^7^ exosome particles.

The exosomes were also quantitated using a nanoparticle tracking analysis system (NTA, Malvern Pananalytical Ltd., Malvern, UK) equipped with a 488 nm blue laser, syringe pump, and CMOS camera. Exosome samples were thawed at RT immediately prior to the analysis and diluted 1:1000 in 1× PBS. The samples were introduced using a syringe and captured at ambient temperature. Background measurements were taken using filtered PBS, which did not reveal the presence of any kind of particle.

### 2.7. Differentiation of MEFs to the Pancreatic Lineage Using Insulinoma-Derived Exosomes

Our reprogramming protocol is divided into three stages: Stage 1, MEF to pancreatic endoderm; stage 2, pancreatic endoderm to pancreatic progenitors; and stage 3, pancreatic progenitors to β-like cells. 5 × 10^4^ MEFs/well were seeded in 12 well tissue culture plates in DMEM containing 10% FBS and 1× P/S for 1 day. The next day, stage 1 differentiation medium was added. The stage 1 differentiation medium contained 1 μM Bix-01294 (MedchemExpress, Monmouth Junction, NJ, USA), 280 μM 2-phospho-L-ascorbic acid (pVc, Sigma Aldrich, Inc., Saint Louis, MO, USA), and 50 ng/mL activin A (R&D Systems, Minneapolis, MI, USA), and cells were kept in it for 6 days. The spent medium was changed every third day. Exosomes were administered twice in stage 1 medium at an interval of 3 days. At the end of 6 days, stage 2 differentiation medium was added for 4 days. It contained four small molecules: 0.5 nM TTNPB (MedchemExpress, Monmouth Junction, NJ, USA), 1 μM repsox (MedchemExpress, Monmouth Junction, NJ, USA), 2 μM cyclopamine (Tocris, Bristol, UK), and 280 μM pVc. The stage 3 medium contained the following components: 1 µM SB203580 (MedchemExpress, Monmouth Junction, NJ, USA), 1× insulin-transferrin-selenium (ITS, Gibco, Thermo Fisher Scientific, Inc., Waltham, MA, USA), 10 mM nicotinamide (Sigma Aldrich, Inc., Saint Louis, MO, USA), 1 µg/mL laminin (Sigma Aldrich, Inc., Saint Louis, MO, USA), 50 ng/mL Exendin-4 (MedchemExpress, Monmouth Junction, NJ, USA), 2 µM Bay K-8644 (Tocris, Bristol, UK), 1× B27 plus supplement (Gibco, Thermo Fisher Scientific, Inc., Waltham, MA, USA), and pVc, and cells were kept in it for 10 days.

Complete knockout DMEM was used as the basal differentiation medium (media control), and contained 15% knockout serum replacement (Gibco, Thermo Fisher Scientific, Inc., Waltham, MA, USA), 5% FBS (exosome depleted), 1% Glutamax (Gibco, Thermo Fisher Scientific, Inc., Waltham, MA, USA), 1% non-essential amino-acid (NEAA, Gibco, Thermo Fisher Scientific, Inc., Waltham, MA, USA), and 0.5 mM β-ME (Sigma Aldrich, Inc., Saint Louis, MO, USA).

### 2.8. Gene Expression Analysis Using Quantitative Reverse-Transcription Polymerase Chain Reaction (qRT-PCR)

Total cellular RNA was extracted using a RNeasy Midi Kit (QIAGEN, Hilden, Germany) according to the manufacturer’s instructions. Complementary DNA (cDNA) was synthesized from 1 µg of total RNA using a PrimeScript 1st Strand cDNA Synthesis Kit (Takara Bio, Inc., Shiga, Japan). A pancreatic lineage-specific gene expression analysis was performed with iQ SYBR Green Supermix (Bio-Rad Laboratories, Inc., Hercules, CA, USA) in a real-time PCR machine (CFX, Bio-Rad Laboratories, Inc., Hercules, CA, USA). The primers used in this study are listed in Supplementary Table S1.

### 2.9. Western Blotting to Detect Exosome-Associated Proteins

Western blotting was performed as previously described [18]. Briefly, the exosome samples were lysed using RIPA buffer, and protein quantification was carried out using a Pierce BCA kit according to the manufacturer’s protocol. 50 µg of protein from MIN6-derived cell lysate and exosomes were resolved using sodium dodecyl sulphate (SDS)-polyacrylamide gel electrophoresis, transferred to polyvinylidene fluoride membranes, and probed with primary antibodies (Supplementary Table S2). The blots were then exposed to secondary antibodies (mouse/rat; Jackson Immunoresearch Laboratories, West Grove, PA, USA). The proteins were detected using an enhanced chemiluminescence reagent (ELPIS Biotech, Daejon, Korea) and visualized by exposure to X-ray film.

### 2.10. Isolation of Mouse Exocrine Cells

Exocrine tissue was harvested from three 8-week-old male mice (C57BL/6, Orient Bio, Inc., Gyeonggi-do, Korea), as reported earlier [19]. All mouse experiments were done using institutional regulations and the study was approved by the Animal Care Committee, School of Medicine, Sungkyunkwan University. Briefly, mouse pancreata were injected with filtered 0.8 mg/mL of collagenase P (Merck KGaA, Darmstadt, Germany) and incubated at 37 °C in a water bath for 15 min. All the exocrine cells were separated from the islets using density gradient centrifugation in Biocoll separation solution (Merck KGaA, Darmstadt, Germany). The cells were centrifuged at 2000 rpm for 20 min at 20 °C. The acinar and ductal cells (from their respective gradient layers) were collected and filtered through a 70 µm mesh (Corning, Sigma Aldrich, Inc., Saint Louis, MO, USA). The cells were finally seeded in RPMI 1640 medium supplemented with 10% FBS and 1× P/S.

### 2.11. The Differentiation of Mouse Exocrine Cells into β-Like Cells Using MIN6-Derived Exosomes

The exocrine cells were seeded in stage 3 differentiation medium in 6 well plates pre-coated with a 1:100 dilution of Matrigel (BD Biosciences, CA, USA). After 1 day, 50 µg of MIN6-derived exosomes were added in stage 3 medium to one well but not the other, and both wells were incubated for 6 days, with a media change every 3rd day (exosomes added every 3rd day in that condition). RNA isolation was performed on cells harvested on the 7th day, followed by qRT-PCR.

### 2.12. Transfection of MEFs and 266-6 Cells

MEFs (5 × 10^4^ cells/well of a 12 well plate) were transfected using jetMESSENGER (Polyplus-Transfection, Illkirch, France) reagent according to the manufacturer’s instructions. Briefly, specific miRNAs (200 nM) (QIAGEN, Hilden, Germany) were diluted in mRNA buffer, and 2 µL of jetMESSENGER reagent was added. The mixture was incubated for 20 min at RT and added to MEFs, and then after 24 h, fresh stage-specific medium was added. The 266-6 cells (8 × 10^4^ cells/well) were transfected using RNAiMAX reagent (Thermo Fisher Scientific, Inc., Waltham, MA, USA) according to the manufacturer’s protocol. Transfection was conducted in stage-specific medium, and the transfected cells were incubated for the desired time.

### 2.13. miRNA Quantitation by qRT-PCR

For miRNA quantification, miRNAs from the MIN6-derived exosomes and their parent cells were isolated using an miRNeasy Serum/Plasma Kit (QIAGEN, Hilden, Germany) and mirVana miRNA isolation kit (Thermo Fisher Scientific, Inc., Waltham, MA, USA), respectively, according to the manufacturers’ instructions. The isolated small RNAs (enriched in miRNAs) were quantified using a Nanodrop spectrophotometer (Nanodrop Technologies, Thermo Fisher Scientific, Inc., Waltham, MA, USA). An equal amount of RNA was subjected to cDNA synthesis using a miRCURY LNA RT Kit (QIAGEN, Hilden, Germany), and expression was detected using a miRCURY LNA SYBR Green PCR Kit (QIAGEN, Hilden, Germany) with U6 snRNA as the control. Predefined primer sets (miRCURY LNA miRNA PCR assays, QIAGEN, Hilden, Germany) corresponding to each of the respective miRNAs were used to perform the qRT-PCR.

### 2.14. miRNA Profiling of MIN6-Derived Exosomes and MIN6 Cells

Total RNA, including small RNA, was converted into cDNA libraries using a SMARTer smRNA-Seq Kit for Illumina (Takara Bio, Inc., Shiga, Japan) following the manufacturer’s protocol. All the cDNA libraries were checked for quality (Bioanalyzer 2100, MIN6-derived exosome-based library, and Tape Station, MIN6 cell-based library, Agilent Technologies, Santa Clara, CA, USA) and fluorimetrically quantified using PicoGreen (Nanodrop Technologies, Thermo Fisher Scientific, Inc., Waltham, MA, USA). The cDNA fragments were sequenced on the Illumina HiSeq2500 platform.

After sequencing, the raw sequence reads were filtered for quality. The adapter sequences were trimmed from the raw sequence reads. Both the trimmed reads and non-adapter reads were considered as processed reads and used for analyzing long targets (≥50 bp). The processed reads formed a unique cluster of reads that 100% matched the sequence identity and read length. To eliminate the effect of large amounts of ribosomal RNA (rRNA) from this study, the reads were aligned to the rRNA sequence, and matching reads were discarded. The remaining reads (devoid of rRNA reads) were sequentially aligned to the reference genome, miRBase (Manchester, UK) v21, and the non-coding RNA database, RNAcentral v10.0, to classify the known miRNAs and other types of RNA, such as tRNA, snRNA, and snoRNA. Novel miRNA prediction was performed by miRDeep2. The read counts corresponding to each smRNA were extracted from the mapped smRNAs to determine the abundance of each one. Differentially expressed smRNAs were determined using statistical methods to compare expression across MIN6-derived exosomes and MIN6 cells. The miRNA sequencing result has been submitted in GEO database with accession number GSE159029.

### 2.15. Immunostaining Studies

After completing stage 3 of differentiation, the differentiated β-like cells were harvested and immunostained as described previously [7]. The list of antibodies is provided in Supplementary Table S2. The samples were visualized using a fluorescence microscope (IX71S1F3, Olympus, Tokyo, Japan).

### 2.16. Determination of C-Peptide Release by Differentiated β-Like Cells

2 × 10^5^ MEFs were seeded in 60 mm tissue culture plates (SPL Life Sciences, Pocheon, Korea), and the MEF-to-β-like cell differentiation was initiated as described above. Different small molecules and growth factors were added at different stages (as described in Section 2.7). After completion of stage 3 differentiation, the differentiated cells were harvested and washed three times with 1× PBS. The C-peptide stimulation followed by ELISA (ALPCO, Salem, MA, USA) analyses were done as described previously [7].

### 2.17. Statistical Analyses

Two-sided, paired student’s *t*-testing was used to determine the statistical significance of differences between conditions. *p* < 0.05 was considered statistically significant. Each experiment was carried out independently three times, and the values are expressed as the mean ± S.E.M. Statistical analyses were conducted using Prism v8.0 (GraphPad Software, Inc., San Diego, CA, USA). For the miRNA sequencing experiment, statistical analysis was performed using Fold Change, exactTest using edgeR per comparison pair. Significant results were deemed to be those that met the conditions of |fc| ≥ 2 and an exactTest raw *p*-value < 0.05.

## 3. Results

### 3.1. Chemical-Based Differentiation of MEF to β-Like Cells

**Figure 1:**
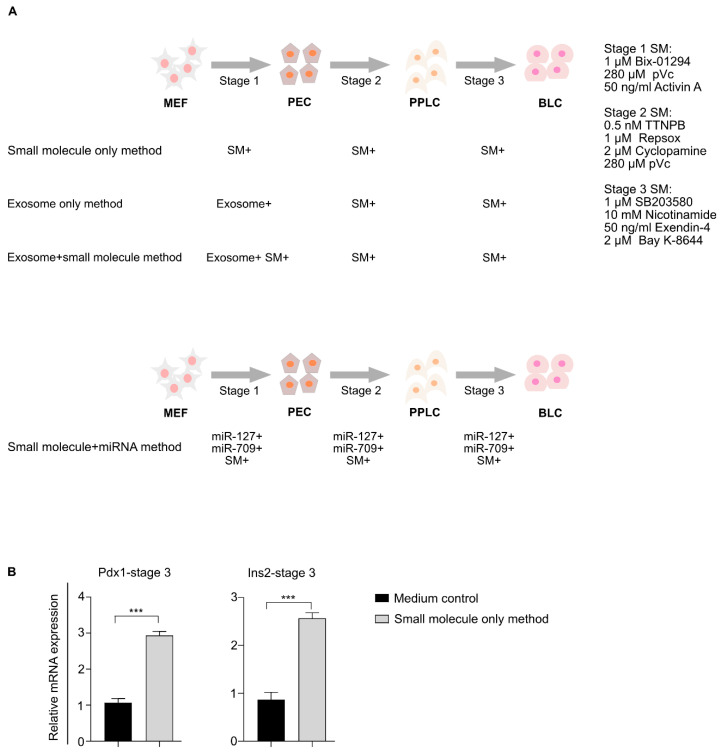
Development of a chemical-based protocol for mouse embryonic fibroblast (MEF) differentiation. (**A**) Schematic diagram representing a new small molecule-based method for differentiating MEFs into β-like cells. Additional methods, including exosome only and exosome + small molecule-based differentiation methods, were also investigated. The exosome-enriched miRNAs + small molecule-mediated differentiation approach is depicted in the lower panel. (**B**) Comparison of pancreatic gene expression in MEFs differentiated using our small molecule only method and medium control cells. *** *p* < 0.001. Statistical significance was determined by paired, two-tailed *t*-testing. The data shown represent the mean ± SEM of three independent experiments. Complete knockout DMEM was used as the basal medium in all differentiation methods. The medium control cells represent cells grown in basal medium. Stage 1, 2, and 3 media consisted of basal medium + stage 1, 2, and 3-specific small molecules. SM = small molecules, SM+ = addition of small molecules, exosome+ = addition of exosomes, exosome+ SM+ = addition of both exosomes and small molecules, miR-127+ = addition of miR-127, miR-709+ = addition of miR-709, MEFs = mouse embryonic fibroblasts, PECs = pancreatic endoderm cells, PPLCs = pancreatic progenitor-like cells, BLCs = β-like cells, pVc = 2-phospho-L-ascorbic acid, and Ins2 = Insulin-2, ns = non-significant.

Previously, it was reported that MEFs could be differentiated into β-like cells using transient expression of four key iPSC factors (Oct4, Sox2, Klf4, and c-Myc) and the addition of small molecules [20]. To generate a protocol for direct differentiation that did not include genetic factors to make it suitable for use in a clinical setting, we developed a chemical-based differentiation method (“small molecule only method”) that can be used to cause normal MEFs, which lack doxycycline-inducible iPSC factors, to differentiate into β-like cells. Our differentiation protocol had three stages modified from previously published protocols [5,20,21]: Stage 1, MEF to pancreatic endoderm cells (PECs); stage 2, PECs to pancreatic progenitor like-cells (PPLCs); and stage 3, PPLCs to β-like cells (BLCs) (Figure 1A).

To confirm the differentiation potential of our small molecule only method, we checked the expression of Pdx1 and Insulin-2 (pancreatic β-cell markers) at the end of the differentiation period (Figure 1B). Our protocol induced 2.9- and 2.6-fold expression of Pdx1 and Insulin-2, respectively, compared with the medium control cells. Thus, the newly generated chemical-based differentiation protocol successfully differentiated MEFs into β-like cells.

### 3.2. Characterization of MIN6-Derived Exosomes

Exosomes are membrane-bound structures that carry a specific message in a functional form from the parent cell to a recipient cell [22], and thus they might have a potential to induce the genes in recipient cells similar to parent cells, which has been proved in several cases [12,14,23]. To confirm our hypothesis of exosome-mediated differentiation to the pancreatic lineage, we used an ultracentrifugation-based method to isolate exosomes from mouse MIN6 insulinoma cell line. Before initiating the differentiation experiments, we characterized these exosomes to confirm that they had conventional exosome-associated features. The TEM images reveal that most of the MIN6-derived exosomes were 200 nm or smaller (Supplementary Figure S1A), and our NTA revealed that the modal size was 157.7 ± 5.3 nm (Supplementary Figure S1B), and that exosome size is in agreement with previously published reports [24,25]. Conventionally, tetraspanins (such as CD63, CD9, and CD81), Alix, and TSG101 proteins are considered to be associated with exosomes. Also, the absence of endoplasmic reticulum-related proteins such as calnexin indicates that the exosome preparation has little to no contamination from cellular remains [18]. Western blot images reveal the enrichment of CD9, Alix, and TSG101 in MIN6-derived exosomes, compared with the MIN6 cell lysate. Also, the absence of calnexin indicates that the exosome isolation procedure yielded substantially pure exosomes (Supplementary Figure S1C).

### 3.3. Internalization of the MIN6-Derived Exosomes by the MEFs

**Figure 2:**
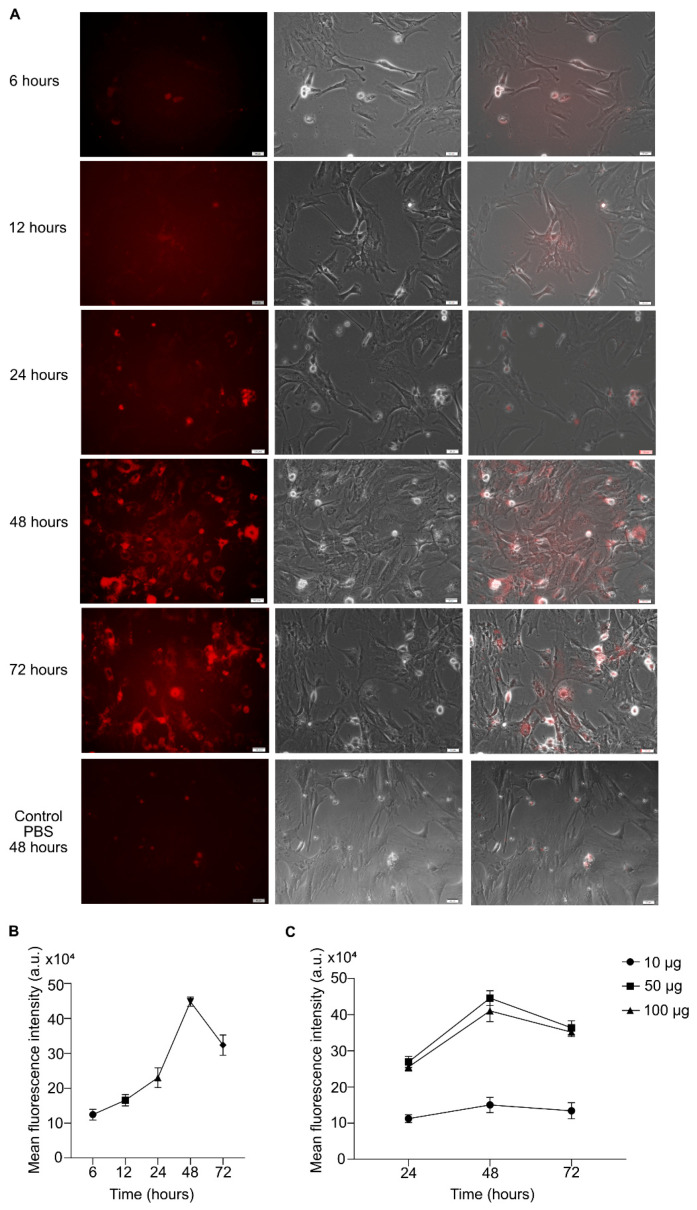
Internalization of the labeled mouse insulinoma (MIN6)-derived exosomes by the MEFs. (**A**) Fluorescence microscopy images depicting the time-dependent internalization of ExoGlow-labeled exosomes (red) by MEF cells. Scale bar = 20 µm. The first panel shows MEFs that have successfully internalized the labeled exosomes (red channel); the middle panel depicts the bright-field images of all the cells present in the respective microscopic field, and the last panel represents the merged images. (**B**) Quantification of the mean fluorescence intensity observed in (**A**). (**C**) Quantitation of the optimal exosome amount required for efficient internalization by MEFs. The data shown represent the mean ± SEM of three independent experiments. a.u. = arbitrary units.

Internalization of the exosomes by the recipient cells is required for exosome-dependent cellular differentiation to occur. Therefore, we investigated the internalization of the MIN6-derived exosomes by the MEFs using lipophilic ExoGlow dye (red fluorescence) that specifically labels intact exosome membranes. Only MEFs that successfully internalized the labeled MIN6-derived exosomes emitted intense red fluorescence (Figure 2A). To identify the time during which maximal exosome internalization occurred, so that we could optimize the exosome addition in stage 1 of our protocol, we performed a time-dependent internalization experiment. The fluorescence microscopy images clearly show that exosome internalization started after 6 h (before 6 h, no visible fluorescence from the MEFs could be observed) of incubation with the MEFs. Exosome internalization visibly increased after 24 h, and after 48 h, the maximum number of exosomes had been internalized by the MEFs (the highest mean fluorescence intensity (MFI) was detected at 48 h); thereafter the internalization process decreased (Figure 2A,B). Therefore, we decided to administer the exosomes in our differentiation protocol at an interval of 72 h (twice) during stage 1 (entire stage 1 duration, 6 days). PBS (equal in volume to the MIN6-derived exosomes) was labeled similarly and used as the control. PBS does not contain intact membranous vesicles, so the dye molecules did not stain it; however, the presence of a few remaining free dye molecules in the PBS could not be ruled out (labeling procedure included free dye removal step). Consequently, some of the MEFs incubated with the labeled PBS emitted a low level of background fluorescence that could be detected only after 48 h of incubation (time point corresponding to maximal exosome internalization). Therefore, the observed red fluorescence in the internalization experiments certainly came from MEFs able to internalize the labeled MIN6-derived exosomes (Figure 2A).

To identify the optimal amount of MIN6-derived exosomes required for proper MEF differentiation, we used different amounts of exosomes (based on the BCA assay) and checked the corresponding exosome internalization by the MEFs (based on MFI). The MEFs showed optimal internalization at 50 µg; at 100 µg, the internalization process decreased, probably due to the sheer abundance of the surrounding exosomes, which could have overwhelmed the internalization pathways (Figure 2C). With 10 µg of exosomes, the MEFs were only faintly fluorescent. Therefore, 50 µg of MIN6-derived exosomes could be used for efficient MEF differentiation to the pancreatic lineage without overloading the MEF exosome-internalization machinery.

### 3.4. Exosome-Based Differentiation of MEFs toward the Pancreatic Lineage

**Figure 3:**
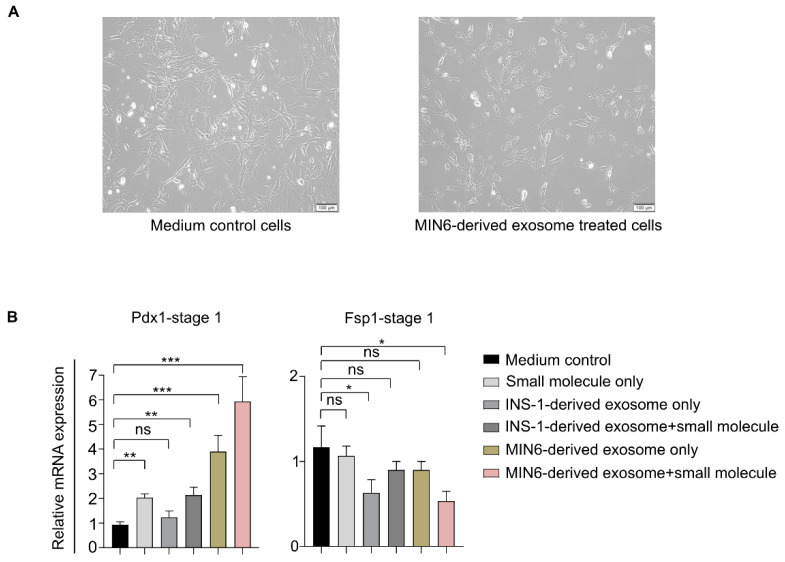
The pancreatic transcriptional program in MEFs treated with insulinoma-derived exosomes. (**A**) Bright-field microscopic images of the medium control and only MIN6-derived exosome-treated MEFs. The insulinoma exosomes (MIN6-derived) did not induce unregulated cellular proliferation in the treated MEFs. Scale bar = 100 µm. (**B**) Relative transcript levels of Pdx1 and Fsp1 in MEFs treated with only small molecules, only exosomes, or the exosome + small molecule combination, compared with the medium control cells in stage 1 of differentiation. * *p* < 0.05, ** *p* < 0.01, and *** *p* < 0.001. Statistical significance was determined by paired, two-tailed *t*-testing. The data shown represent the mean ± SEM of three independent experiments. ns = non-significant. Stage 1 medium consisted of basal medium + stage 1 small molecules. The medium control cells represent cells grown in basal medium.

After confirming the quality and internalization of the purified exosomes, we tested their differentiation potential in MEFs. To test interspecies exosome activity, we also prepared exosomes from the rat insulinoma INS-1 cell line using the same method as for the MIN6-derived exosomes. Because the exosomes might have factors relevant to cancer or β-cells, we tested whether the exosome-treated MEFs underwent unlimited cellular proliferation as cancerous cells do. The exosome-treated cells changed their morphology distinctly from the untreated medium control cells, as seen from the bright-field microscopic images (Figure 3A), but uncontrolled proliferation was not observed. We hypothesized that the insulinoma-derived exosomes would initiate pancreatic endoderm generation, the first step in pancreatic development, in the MEFs because they are derived from pancreatic β-cell lines. Therefore, we performed qRT-PCR to assess the expression of the Pdx1 and Fsp1 genes, the first gene in the pancreatic lineage to be expressed in developing definitive endoderm [26] and a fibroblast-specific marker [16], respectively (Figure 3B), in MEFs grown under various conditions that had completed stage 1: (1) small molecule only method, (2) exosomes only (isolated from MIN6 or INS-1 cells) method, and (3) exosome + small molecule method (Figure 1A). The qRT-PCR results show that the MIN6-derived exosome + small molecule method yielded the highest Pdx1 expression, 5.9-fold compared to the medium control cells (cells grown in basal medium). Cells treated with MIN6-derived exosomes only (exosome only method) expressed a 3.9-fold increase in the Pdx1 transcript level. The small molecule-treated (small molecule only method) cells expressed relatively less (2.0-fold) Pdx1 mRNA, but the amount was still significantly higher than in the medium control cells. The INS-1-derived exosomes only, on the other hand, did not significantly induce Pdx1, probably due to the interspecies barrier between mice and rats. The INS-1-derived exosomes + small molecule combination did induce Pdx1 at a higher level (2.1-fold) than the medium control cells, almost equal to the small molecule only derived Pdx1 transcript expression (Figure 3B). In contrast, Fsp1 gene expression decreased in the MIN6-derived exosome + small molecule-treated MEFs compared with the medium control cells (Figure 3B), suggesting the initiation of MEF differentiation toward another cell type, distinct from their original fibroblastic nature. Therefore, the exosomes definitely contain bioactive molecules that, when combined with specific small molecules, induce Pdx1 expression in the β-cell differentiation protocol that turns on the pancreatic transcriptional program in MEFs.

### 3.5. Pancreatic Marker Expression in Differentiated β-Like Cells

**Figure 4:**
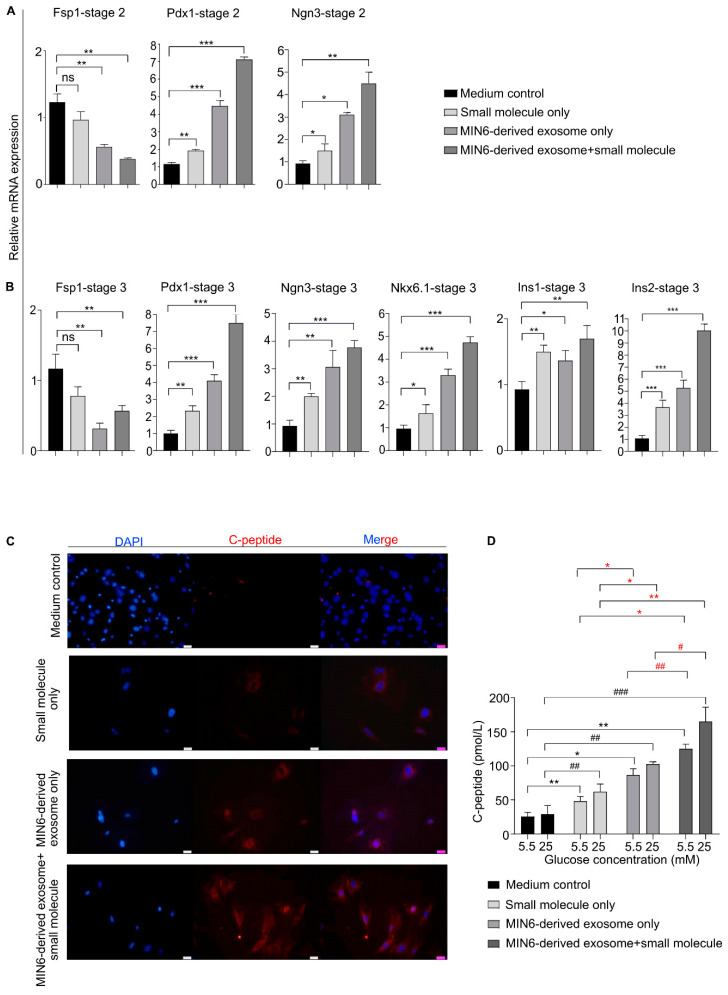
MIN6-derived exosome and small molecule combination enhanced the differentiation of MEFs into β-like cells. (**A**) Relative transcript levels of Fsp1, Pdx1, and Ngn3 in differentiated cells at the end of stage 2. (**B**) qRT-PCR analysis of Fsp1 and different pancreatic genes in differentiated cells after the completion of stage 3. The cells had been exposed to small molecule only, MIN6-derived exosome only, or MIN6-derived exosome + small molecule in stage 1 and then differentiated further. (**C**) Immunostaining for the detection of C-peptide protein in cells grown under different conditions after stage 3. Scale bar = 20 µm. (**D**) C-peptide release assay to determine the functionality of the differentiated post-stage 3 cells grown under different conditions. * *p* < 0.05, ** *p* < 0.01, and *** *p* < 0.001 compared to media control cells exposed to 5.5 mM glucose. # *p* < 0.05, ## *p* < 0.01 and ### *p* < 0.001 compared to 25 mM glucose-challenged media control cells. Statistical significance was determined by paired, two-tailed *t*-testing. The data shown represent the mean ± SEM of three independent experiments. # represents comparisons between the MIN6-derived exosome only and MIN6-derived exosome + small molecule combination conditions. * represents small molecule only versus all other conditions. Ins1 = Insulin-1, Ins2 = Insulin-2, and ns = non-significant. Stage 2 or 3 media consisted of basal medium + stage 2 or 3 small molecules. The medium control cells represent cells grown in basal medium.

We next explored pancreatic gene expression patterns in stages 2 and 3 (Figure 1A). We anticipated that the MEFs that had completed stage 1 of differentiation would undergo further differentiation, ultimately producing pancreatic β-like cells, and therefore, we exposed those cells sequentially to the specific small molecules and growth factors used in other β-cell differentiation protocols. The expression of Pdx1 and Ngn3 in stage 2 of differentiation was quantified using qRT-PCR. We observed high-level expression of Pdx1 in stage 2 cells. As in stage 1, the exosome + small molecule-treated cells expressed the highest level of Pdx1 (6.4-fold compared with the control cells). Ngn3, a transcription factor expressed in endocrine progenitor cells [20,27], was present in the stage 2 cells, with the highest expression (4.8-fold that in the control cells) in the exosome + small molecule condition. The expression of the Fsp1 transcript was consistently low in the differentiated cells (Figure 4A). The cells treated with the MIN6-derived exosomes only expressed significantly higher levels of Pdx1 and Ngn3 mRNA than the cells treated with small molecules only and the medium control cells; however, their gene expression was lower than in the exosome + small molecule condition (Figure 4A).

We next assessed the presence of pancreatic markers in stage 3 cells at the end of differentiation. In rodents, insulin genes are composed of Insulin-1 (derived from the retro-transposition of the partially processed form of the Insulin-2 transcript) and Insulin-2 [28]; human insulin is homologous to Insulin-2 in mice [29]. We observed that the exosome + small molecule-treated cells expressed significantly more Insulin-1 (1.7-fold) than cells in the other conditions (Figure 4B). Similarly, the Insulin-2 mRNA level increased significantly (10.0-fold) compared with the medium control cells (Figure 4B). Insulin-2 mRNA levels were 2-fold higher in the exosome + small molecule condition than in the MIN6-derived exosome only treated cells (Figure 4B). It has been reported that extended culture of mouse-derived insulinoma cell lines such as β-TC3 and MIN6 produces differential Insulin-1 and Insulin-2 expression (decline in Insulin-1 relative to Insulin-2 mRNA) [19]. Therefore, the differential expression pattern of Insulin-1 and Insulin-2 in the differentiated MEFs could be a consequence of the prolonged culture of the MIN6 cells from which the exosomes were derived. That differential expression between Insulin-1 and Insulin-2 might also have been reflected in the contents of the MIN6-derived exosomes. The expression of Nkx6.1 was significantly higher (4.8-fold) in the exosome + small molecule-treated cells than in cells in the other conditions (Figure 4B). Nkx6.1 expression in pancreatic progenitors is known to suppress the development of endocrine cells other than β-cells, and it helps in maintaining β-cell identity [30]. Ngn3 expression, on the other hand, declined compared with stage 2 (Figure 4A,B), which is in accordance with the developmental pattern of Ngn3 gene expression; Ngn3 expression is highest in endocrine progenitors, and after endocrine commitment, Ngn3 levels decline [27]. Thus, we propose that the exosomes differentiated the MEFs into PECs, and the inclusion of specific small molecules in a stage-wise manner augmented the differentiation process, leading to enhanced expression of pancreatic β-cell genes in the differentiated β-like cells. Supplementary Table S3 describes changes in gene expression pattern.

To confirm cellular differentiation in terms of protein expression, Pdx1, Nkx6.1, and C-peptide levels were checked by immunostaining. As seen in the images, the highest expression of Pdx1, Nkx6.1, and C-peptide occurred in the exosome + small molecule combination. The cells treated with exosomes only also showed expression of those proteins but at lower levels (Supplementary Figure S2A,B and Figure 4C). The small molecule only treated cells were faintly positive for Pdx1, Nkx6.1, and C-peptide (Supplementary Figure S2A,B and Figure 4C). However, no Pdx1, Nkx6.1, or C-peptide positive cells were observed in the medium control cells (Supplementary Figure S2A,B and Figure 4C). We further quantified the Pdx1, Nkx 6.1, and C-peptide protein levels (Supplementary Figure S2C,D) under different culture conditions in stage 3 differentiated cells, which additionally supports our previous results based on mRNA expression, protein levels (immunostaining) and C-peptide ELISA.

We wanted to further investigate the functionality of the differentiated β-like cells. Functional β-like cells maintain euglycemia by secreting insulin in response to increased blood glucose levels [2]. There are many methods that are used to assess β-cell functionality in vivo, which includes the usage of hyperglycemic clamp, intravenous and oral glucose tolerance test, fasting proinsulin to insulin (or C-peptide) ratio [31]. In vitro methods used for assessing β-cell function includes glucose uptake, insulin or C-peptide ELISA [5,32,33]. Because we had included an insulin-transferrin-selenium (ITS) supplement in stage 3 of differentiation, the detection of C-peptide instead of insulin was preferred because cells sometimes take up insulin passively from the culture medium [6]. Furthermore, it is also well established that the presence of C-peptide in the cells indicates active insulin synthesis in the differentiated β-cells [5,34,35]. Therefore, we measured the released C-peptide by ELISA to assess the functionality of β-like cells. We treated stage 3 differentiated cells grown under various conditions to sequentially low and high glucose challenges to assess their glucose responsiveness. The cells were exposed to 5.5 mM glucose (low glucose) for 1 h, and the C-peptide released in the medium indicated the basal level of insulin released. The same cells were then exposed to 25 mM glucose (high glucose), and the C-peptide released reflected glucose-stimulated insulin secretion. The cells treated with exosomes + small molecules, exosomes only, and small molecules only secreted an average of 145.4, 90, and 48.5 pmol/L of C-peptide, respectively, upon exposure to 5.5 mM glucose (Figure 4D). When challenged with 25 mM glucose, the same cells released an average of 180, 112.5, and 77 pmol/L of C-peptide, respectively (Figure 4D). The C-peptide ELISA experiment revealed that the exosome + small molecule-treated cells released the highest C-peptide level under low and high glucose conditions (Figure 4D). The cells grown using the exosome only and small molecule only methods were also able to respond to the glucose challenges, with the former responding better than the latter, compared to the medium control cells (Figure 4D). In other words, the exosome + small molecule-treated cells not only induced the highest pancreatic gene expression but also expressed Pdx1, Nkx6.1, and C-peptide proteins at significantly higher levels at the end of the differentiation period and responded well to glucose challenges, thus exhibiting enhanced differentiation to β-like cells compared with cells in the other culture conditions.

### 3.6. Lineage Tracing Revealed the Fibroblastic Origin of the Differentiated β-Like Cells

To eliminate the possibility that contaminating endocrine progenitors were present in the starting MEF cells and confirm that the differentiated β-like cells indeed originated from the MEFs, we performed a lineage tracing experiment in a way similar to that previously reported [36]. We crossed Fsp1-cre and R26RtdTomato mice to acquire Fsp1-tdTomato offspring in whom tdTomato expression was under the control of the Fsp1 promoter. Therefore, only cells originating from fibroblasts expressed tdTomato (red fluorescence) (Supplementary Figure S3A). tdTomato-MEFs were harvested [36] and the cells were divided into small molecule only, exosome only, and exosome + small molecule groups in stage 1 and differentiated according to our protocol (Figure 1A). At the end of stage 3, the cells were stained to assess their C-peptide protein levels. The exosome + small molecule combination yielded the highest number of C-peptide-positive cells among the groups (Supplementary Figure S3B–D). Also, the differentiated cells emitted red fluorescence, indicating that they originated from the tdTomato-MEFs. The co-expression of the C-peptide and tdTomato proteins in cells differentiated under various conditions suggests that these cells originated from tdTomato-positive MEFs, and the diverse differentiation conditions induced them to express C-peptide at various levels. The medium control cells did not express any C-peptide, but they did express tdTomato protein (Supplementary Figure S3E). Therefore, using our culture conditions, the MEFs undeniably undergo differentiation to give rise to β-like cells.

### 3.7. Differentiation of Mouse Exocrine Cells into β-Like Cells Using MIN6-Derived Exosomes

**Figure 5:**
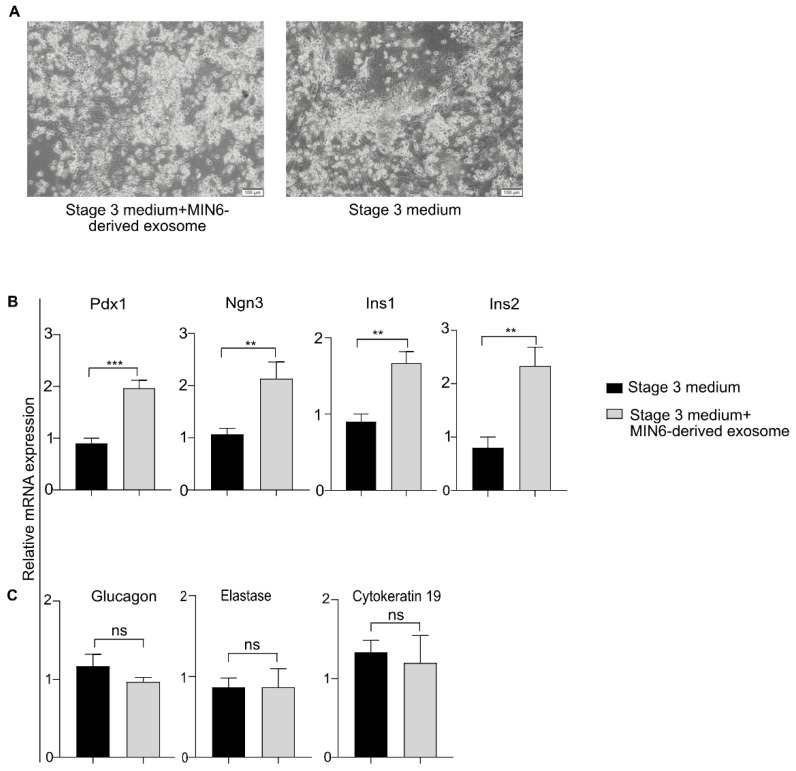
Differentiation of primary mouse exocrine cells into β-like cells using MIN6-derived exosomes. (**A**) Bright-field images of exocrine cells grown in stage 3 medium with or without 50 µg of MIN6-derived exosomes. Scale bar = 100 μm. (**B**) qRT-PCR analysis of pancreatic β-cell markers. (**C**) Gene profiling of α, acinar, and ductal cell-specific genes using qRT-PCR. Data are expressed as the mean ± SEM of 3 independent experiments. ** *p* < 0.01, *** *p* < 0.001 (student’s *t*-test) between groups. Ins1 and Ins2 refer to the Insulin-1 and Insulin-2 transcripts, respectively. ns = non-significant. Stage 3 medium consisted of basal medium + stage 3 small molecules and growth factors and supplements.

Because the exocrine and endocrine cells of the pancreas share a common developmental pathway [37], we anticipated that the MIN6-derived exosomes could directly differentiate primary mouse exocrine cells into β-like cells. Because the MIN6-derived exosomes carry a β-cell-specific message, we assumed that they would repress the exocrine (acinar and ductal cell) transcriptional program in differentiating mouse exocrine cells. Furthermore, the exocrine cells had already acquired a pancreatic fate, and thus we hypothesized that exocrine cells could be differentiated by treatment with stage 3 medium and exosomes (omitting stages 1 and 2). To test that hypothesis, we isolated exocrine cells from the pancreata of 8-week-old mice and added the stage 3 medium containing exosomes to the isolated cells. As seen from the bright-field microscopic images, there was no apparent morphological difference between the exocrine cells exposed to stage 3 medium in the presence or absence of MIN6-derived exosomes (Figure 5A). The qRT-PCR analysis revealed that the levels of Pdx1 and Ngn3 increased by 2.1- and 2.0-fold, respectively, in the exocrine cells treated with MIN6-derived exosomes compared with the non-treated cells (Figure 5B). The Insulin-1 and Insulin-2 transcripts increased by 1.8- and 2.9-fold, respectively (Figure 5B) in the exosome-treated versus non-treated exocrine cells. On the other hand, the expression of glucagon (α-cell marker) [38], elastase (acinar cell marker) [37], and cytokeratin19 (ductal cell marker) [39] was similar in both the exosome-treated and non-treated exocrine cells (Figure 5C). Pdx1 is the first transcription factor to be expressed by PECs and is also present in mature β-cells [26]. Pdx1 is also reported to be expressed during the generation of ductal epithelium; however, its expression is lost in mature duct cells [40]. Similarly, acinar cells lose Pdx1 expression with maturation [40,41]. During development, endocrine cells, including β-cells, arise from Ngn3-positive progenitor cells, and Ngn3 is both sufficient and necessary for the commitment to the endocrinal lineage [27,37]. Therefore, the insulinoma (MIN6)-derived exosomes transmitted the β-cell-specific signal to the exocrine cells, as observed from the increased Insulin-1 and Insulin-2 transcript levels. Moreover, MIN6 is a β-cell line, therefore, it is unlikely that the MIN6-derived exosomes would carry α-(glucagon-specific), acinar-(elastase-specific), or ductal-(cytokeratin-specific) message. Therefore, no increase in glucagon (a marker for α-cells, another islet cell type) or acinar or ductal cell marker expression could be observed in the exosome-treated cells. In conclusion, the MIN6-derived exosomes were able to induce the β-cell transcriptional program in primary mouse exocrine cells.

### 3.8. Identification of the miRNA Profile of the MIN6-Derived Exosomes

Exosomes carry various biomolecules, including miRNAs [9], that are known to regulate gene expression during organismal development [42]. Therefore, we investigated the miRNAs present in the MIN6-derived exosomes by performing miRNA sequencing (miRNA-seq) to identify which miRNAs were mainly responsible for MEF differentiation. The miRNA-seq assay revealed the differential expression of miRNAs between the exosomes and the parental MIN6 cells, as demonstrated by the heatmap diagram in Supplementary Figure S4, possibly through the active sorting mechanisms operative during exosome biogenesis. In MIN6-derived exosomes, 131 miRNAs were upregulated 2-fold higher (*p* < 0.05) than in the MIN6 parent cells, and 121 miRNAs were 2-fold downregulated (*p* < 0.05) (Supplementary Table S4).

The top 20 upregulated miRNAs and their respective fold changes between MIN6-derived exosomes and MIN6 cells are shown in Supplementary Table S5. Of all the miRNA-seq-identified miRNAs, mmu-miR-486a/b-5p showed the highest abundance (616-fold) in exosomes compared with MIN6 cells. miR-486-5p is reported to be expressed in many cancers, including pancreatic cancers, where it controls invasion and metastasis [43]. Given the cancerous phenotype of MIN6 cells, the enrichment of miR-486-5p in the MIN6-derived exosomes is not surprising. Other upregulated miRNAs are involved in pancreatic development, such as mmu-miR-19b-3p (179-fold), mmu-miR-127-5p (104-fold), mmu-miR-709 (97-fold), and mmu-miR-494-3p (33-fold). The expression of many miRNAs remained unaltered between exosomes and their parent MIN6 cells (Supplementary Table S6). For example, The expression of many miRNAs remained unaltered between exosomes and their parent MIN6 cells like mmu-miR-30a-5p (1.2-fold upregulated), mmu-miR-484 (1.12-fold upregulated), and mmu-miR-690 (1.04-fold upregulated).

Many miRNAs were found to be predominantly present in MIN6 cells compared with the exosomes, as shown by the miRNA-seq data (Supplementary Figure S4 and Table S4). For example, miR-375-5p was enriched in MIN6 cells compared with the MIN6-derived exosomes, which is contradictory to a previous report [44]. The variability could be a consequence of the different MIN6 culture conditions and exosome isolation methods used [45] in the two studies. The let-7 family was also found to be upregulated in the MIN6 cells compared with the exosomes in our miRNA-seq experiment.

### 3.9. Validation of the miRNA-seq Data Using qRT-PCR Analysis

**Figure 6:**
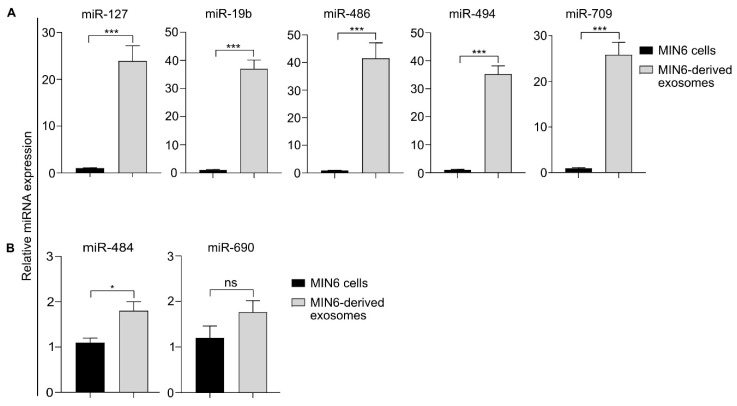
Relative expression of miRNAs inside exosomes and parent cells. The expression of (**A**) highly upregulated miRNAs (miR-127, mir-19b, miR-486, miR-494, and miR-709) is shown, and the expression of (**B**) non-moderated miRNAs (miR-484 and miR-690) is compared with that of the U6 snRNA control. Significant differences are indicated by * *p* < 0.05 and *** *p* < 0.001. Statistical analysis was performed using two-tailed, paired *t*-testing of three independent experiments. ns = non-significant.

To validate the robustness of the data generated using miRNA-seq, qRT-PCR analyses were performed on miRNAs isolated from the MIN6 cells and exosomes. Based on the fold change values, we investigated both upregulated (in exosomes) and non-moderated (equal representation in both exosomes and cells) miRNAs from the miRNA-seq data. The expression level of miR-486a/b-5p (miR-486) was 48.8-fold higher in the exosomes than in the MIN6 cells. Similarly, miR-127-5p (miR-127), miR-19b-3p (miR-19b), miR-494-3p (miR-494), and miR-709 were expressed 23.2-, 37-, 35.2-, and 26.9-fold more, respectively, in the exosomes than in the parent cells (Figure 6A). On the other hand, miR-690, and miR-494 were expressed 1.4- and 1.6-fold more, respectively, in the exosomes than in the parent cells (Figure 6B). Therefore, the miRNA expression pattern identified in the miRNA-seq data comparing MIN6 cells with MIN6-derived exosomes matches well with the miRNA transcript abundance detected using qRT-PCR.

### 3.10. Treating MEFs with miRNAs Upregulated in the miRNA-seq Analysis

**Figure 7:**
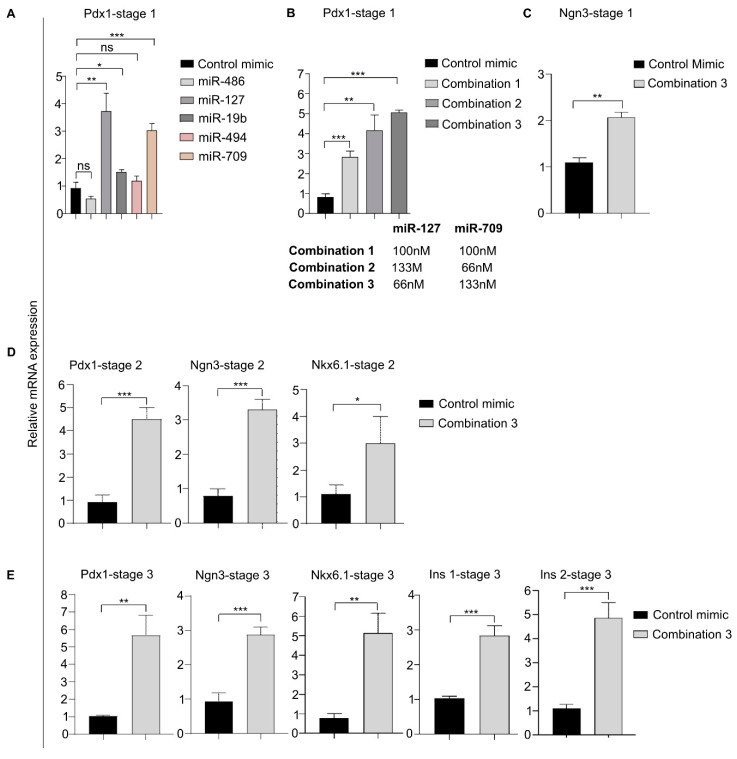
Exogenous addition of upregulated miRNAs to differentiate MEFs into β-like cells. (**A**) Pdx1 gene expression in stage 1 cells after individual transfection with the upregulated miRNAs. (**B**) Assessment of how different miR-127 and miR-709 combinations induce Pdx1 expression in stage 1 cells. (**C**) Ngn3 transcript levels were identified in stage 1 cells after miR-127 and miR-709 transfection using combination 3. (**D**) Pancreatic gene expression profile (qRT-PCR) in stage 2 cells transfected with miR-127 and mir-709 (combination 3). (**E**) qRT-PCR analysis of pancreatic β-cell markers in stage 3 cells transfected with miR-127 and miR-709 (combination 3). * *p* < 0.05, ** *p* < 0.01, and *** *p* < 0.001. Statistical significance was determined by paired, two-tailed *t*-testing. The data shown represent the mean ± SEM of three independent experiments. Ins1 = Insulin-1, Ins2 = Insulin 2, and ns = non-significant. Stage 1, and 2 media consisted of basal medium + stage 1, and 2 small molecules. Stage 3 medium comprised of basal medium + stage 3 small molecules, growth factors and supplements.

We observed that miR-486, miR-127, miR-19b, miR-494, and miR-709 were highly enriched in the MIN6-derived exosomes and are relevant to the pancreatic lineage [46,47]. Therefore, we investigated whether those miRNAs would enhance the expression of pancreatic β-cell markers, individually or in combination. Before transfecting the MEFs with miRNA mimics to simulate naturally occurring miRNAs, we optimized our transfection experiments using a 5′ FAM-labeled (fluorescein amidites; equivalent to fluorescein dye) control miRNA mimic (control mimic had no homology to mouse, rat, or human miRNAs). We performed dose-dependent transfection experiments and found that a dose of 200 nM gave approximately 80% transfection efficiency with low cytotoxicity (Supplementary Figure S5A). Next, we performed individual transfections to identify the miRNAs that induced higher levels of Pdx1 in stage 1 because we intended to mimic the exosome-mediated differentiation in stage 1 (Figure 7A). We found that miR-127 and miR-709 induced Pdx1 expression to the greatest extent (3.9- and 3.2-fold, respectively, greater than the control mimic), followed by miR-19b (1.6-fold). We then performed combinatorial miRNA transfections to partially mimic the function of the miRNA present in the MIN6-derived exosome. We tried several combinations of miR-127, miR-709, and miR-19b; however, none of them could induce higher Pdx1 expression than the individual transfections (Supplementary Figure S5B). The different combinations of miR-127, miR-19b, and miR-709 increased cell proliferation (Supplementary Figure S5C, upper panel), similar to the control group (control mimic transfection). We also noted that when miR-19b was transfected alone, it increased cellular proliferation, unlike individual transfections of miR-127 and miR-709 (Supplementary Figure S5C, lower panel). Therefore, we concluded that miR-19b has a dominating effect on miR-127 and miR-709 such that, when they are transfected together, the cells start proliferating rapidly instead of differentiating. Consequently, pancreatic gene expression was not induced properly when the three were transfected together. Next, we assessed the combination of miR-127 and miR-709 and found that it increased pancreatic gene expression (Figure 7B).

Because the highest Pdx1 transcript level was expressed in stage 1 after miR-127 + miR-709 transfection (combination 3, Figure 7B), we checked whether any other pancreatic gene was induced as a result of the exogenous miRNA. Surprisingly, we detected Ngn3 expression (Figure 7C), though at a low level, in the stage 1 cells. No other pancreatic gene transcript could be detected in the qRT-PCR analysis (data not shown). Next, we explored the pancreatic gene expression pattern in stage 2 cells after transfection with miR-127 + miR-709 (combination 3). We found increased Pdx1, Ngn3, and Nkx6.1 transcripts, with 4.8-, 4.1-, and 2.7-fold expression, respectively, compared to the control group (Figure 7D). We investigated the levels of the pancreatic markers in stage 3 cells using a gene expression analysis and found increased expression of Pdx1 (5.4-fold), Ngn3 (2.8-fold), Nkx6.1 (6.2-fold), Insulin-1 (2.7-fold), and Insulin-2 (4.3-fold) in cells treated with miR-127 + miR-709 (combination 3) compared with the control mimic group (Figure 7E). Thus, we observed that the stage-wise combinatorial treatment of miR-127 and miR-709 enhanced the differentiation of MEFs in the presence of small molecules, establishing our final protocol for differentiating MEFs into β-like cells (Figure 1A, lower panel).

We also compared the pancreatic gene expression in the exosome-treated cells with the miRNA-treated cells, miR-127+709 (combination 3) and miR-127+709+19b (combination 1). We found that the exosome treatment in stage 1 induced highest Pdx1 expression followed by miR-127+709 and miR-127+709+19b. The result is not surprising as the exosomes are rich in proteins including transcription factors, and mRNAs apart from miRNAs (Supplementary Figure S6 and Table S7).

### 3.11. Treatment of Exocrine Cells with Upregulated miR-127 + miR-709

**Figure 8:**
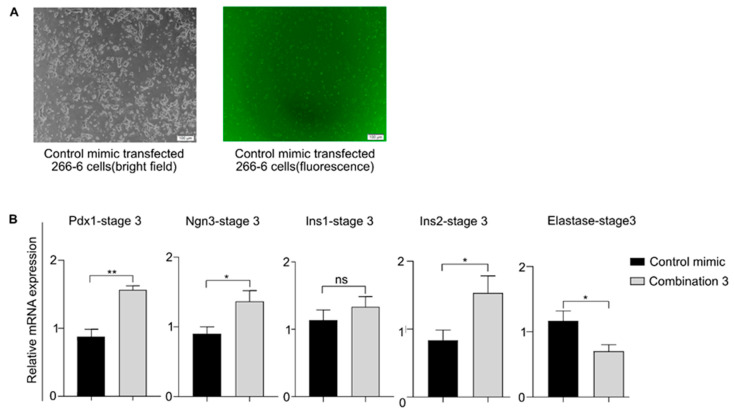
Transfection of miR-127 and miR-709 induces pancreatic β-cell gene expression in acinar cells. (**A**) Transfection optimization in 266-6 cells (acinar cell line) in stage 3 medium. The first panel represents a bright-field image of the 266-6 cells 48 h after transfection, and the second panel is the corresponding green channel (fluorescence microscope) image. Scale bar = 100 µm. (**B**) The acinar cell line (266-6) was transfected with miR-127 + miR-709 (combination 3), which increased the transcript levels of Pdx1, Ngn3, and Insulin-2. The combination decreased the acinar marker elastase expression. * *p* < 0.05 and ** *p* < 0.01. Statistical significance was determined by paired, two-tailed *t*-testing. The data shown represent the mean ± SEM of three independent experiments. Ins1 = Insulin-1, Ins2 = Insulin 2, and ns = non-significant. Stage 3 medium comprised of basal medium + stage 3 small molecules, growth factors and supplements.

We had previously observed that the MIN6-derived exosomes could induce a β-like cell transcriptional program in primary mouse exocrine cells. Next, we tested the effect of miR-127 + miR-709 (combination 3) on a mouse acinar cell line, 266-6. Initially we tried transfecting primary mouse exocrine cells; however, due to poor transfection efficiency, we instead transfected 266-6 cells. The transfection efficiency of the 5′ FAM-labeled control mimic into 266-6 cells was 70–80% (Figure 8A). The transfected 266-6 cells were grown in stage 3 medium to imitate the exosome-mediated effect on primary exocrine cells exposed to stage 3 of differentiation, and pancreatic β-cell marker expression was checked. Consistent with previous results, we found increased expression of β-cell marker genes (Figure 8B). That result corroborated the gene expression pattern induced in the exosome-treated primary exocrine cells. Taking our results together, we thus conclude that the miR-127 and miR-709 present in the MIN6-derived exosomes and their combinatorial effects induce both MEFs and exocrine cells to undergo pancreatic β-like cell differentiation.

## 4. Discussion

Stem cell-based cell therapy holds great promise in the field of regenerative medicine. However, the clinical application of those cells, especially ESCs and iPSCs, is hampered by ethical and tumor induction issues, respectively. Therefore, alternative methodologies, such as direct differentiation using small molecules, are being currently considered for clinical transplantation [4]. Previous studies have used ESC- [21,48], iPSC- [49], and adult stem cell-like MSC-based [6] differentiation protocols for β-like cell generation in vitro. Some studies have instead used the virus-mediated overexpression of different pancreatic genes, such as Pax4 or Pdx1, and MafA or Pdx1, Ngn3, and MafA for the differentiation of δ-, α-, or exocrine-cells into β-like cells, respectively, in vivo [50,51]. MEFs were shown to differentiate into β-like cells upon transient expression of iPSC factors and small molecules [20]. To enable clinical translation and use the potential of exosomes in mediating cellular differentiation, we investigated the ability of insulinoma exosomes to differentiate somatic MEFs into β-like cells. For this purpose, we added exosomes during the initial stage of differentiation to provide pancreatic lineage-specific signals to the starting MEFs and specifically induce them toward a pancreatic fate [5]. We expected that adding exosomes carrying β-cell-specific signals in the presence of an epigenetic modifier such as Bix-01294 [20] and small molecules such as pVc and activin A (a definitive endoderm (DE) inducer) [20,48] in stage 1 would induce the efficient differentiation of MEFs into pancreatic endoderm cells via DE formation. We observed that MIN6-derived exosomes initiated the pancreatic transcriptional program by inducing Pdx1 and repressing Fsp1 expression (Figure 3B). Notably, the exosome + small molecule combination yielded the highest Pdx1 expression among conditions (exosome only, small molecule only, and medium control cells) (Figure 1A and Figure 3B). Furthermore, we observed increased expression of different β-cell genes using qRT-PCR in the cells differentiated under various conditions at the end of stages 2 and 3, especially in exosome-treated cells in the presence or absence of small molecules (exosome treatment was done in stage 1 only) (Figure 4A,B). The exosome + small molecule and exosome only treated cells showed higher Pdx1, Nkx6.1, and C-peptide protein levels and were highly responsive to glucose-mediated stimulation (functional response) compared to cells in other conditions (Figure 4D). As a summary, the expression fold change of the makers for β-cell and MEFs are shown at a glance in Supplementary Table S3. Which clearly showed that the expression levels of the β-cell markers were increased while the expression of the MEF marker was reduced as the differentiation progress. In addition, we confirmed that MIN6-derived exosome + small molecule combination resulted in the highest pancreatic marker expression enhancement. Moreover, the exosome-mediated differentiation of primary mouse exocrine cells also induced β-cell-specific genes (Figure 5B).

Because miRNAs play a major role during pancreatic development, including β-cell generation and function [42], we performed miRNA-seq to explore the miRNA content of the exosomes (Supplementary Figure S4 and Table S4) and investigated how well a few exosome-enriched miRNAs induced pancreatic differentiation in MEFs. We found that the combined treatment of miR-127 + miR-709 (combination 3) induced the highest Pdx1 expression among the tested conditions in stage 1 (Figure 7B). Elevated miR-127 expression is reported to suppress β-cell proliferation and insulin secretion [52], on the one hand, but on the other, it stimulates pancreatic progenitor cell differentiation among endocrine cells [53]. Moreover, the overexpression of miR-127 is known to promote mesendoderm generation via nodal signaling [54]. miR-709, a miRNA predominant in pancreatic β-cell-derived exosomes [55], targets different genes in different tissues [47]. For example, in a peripheral nervous system injury, it targets the Egr2, c-Jun, and Sox-2 transcription factors [56], whereas in adipocytes, it mediates glycogen synthase kinase-3β downregulation, which activates Wnt/β-catenin signaling [57]. Therefore, by targeting a distinct set of genes in each differentiation stage of our protocol, miR-709 might promote the eventual differentiation of MEFs into β-like cells. It is possible that the miR-127 present in the exosomes initially primes mesendoderm formation in the MEFs, followed by DE generation that is mediated by activin A [20]. Thereafter, the Pdx1-positive pancreatic endoderm is generated by the combined action of Bix-01294 and pVc [20] and the help of miR-709. However, a detailed study is required to unravel the mechanisms by which miR-127 and miR-709 induce the creation of pancreatic endoderm and the subsequent generation of functional β-like cells derived from MEFs. The transfection of miR-127 + miR-709 (combination 3) into an acinar cell-line (266-6 cells) in stage 3 induced higher pancreatic gene expression than found in the control group (Figure 8B); however, the induction was less than found in stage 3 MEF cells (Figure 7E). These results corroborate the results generated in primary mouse exocrine cells and probably stem from the less than optimal differentiation signals/conditions for exocrine or acinar-to-β-cell differentiation present in our protocol, which was optimized for MEF-to-β-like cell differentiation.

Our final protocol (Figure 1A, lower panel) includes specific small molecules and miRNAs that guide the starting MEF cells to undergo pancreatic lineage commitment, unlike mouse ESCs [21], in which the spontaneous differentiation of embryoid bodies (EBs) is followed by the addition of pancreatic inducers. In another study [48], mouse ESC-derived EBs were differentiated into DEs using activin A, LiCl, and Noggin and then replated in monolayer format for 6 days with extended activin A and Noggin treatment for pancreatic differentiation. Prolonged activin A treatment in monolayer setup has been shown to interfere with optimal pancreatic differentiation, producing a reduced percentage of C-peptide-positive cells [58]. Our protocol, on the other hand, included activin A treatment for only a short time. In another study, iPSCs derived from nonobese diabetic mice were generated using the lentivirus-mediated expression of iPSC factors, which was followed by EB generation and pancreatic induction [49]. Our protocol does not use virus-mediated iPSC factor induction. A recent study used human iPSC lines to generate EBs that were induced toward the pancreatic lineage. During the late stages of differentiation in that study, human-islet-derived EVs were added to induce intracellular C-peptide levels [59]. Our protocol, on the other hand, harnessed the potential of β-cell-specific exosomes to initiate pancreatic lineage commitment during the initial stages of pancreatic differentiation.

We also have compared the expression fold change of key markers in differentiated cells from current studies with the previous studies using mouse iPSC and MEF cells (suspension culture) as starting cells (Supplementary Table S8). Moreover, we have compared the amount of C-peptide released during low and high glucose challenges in our studies and other previous studies (Supplementary Table S9). We have observed that the levels of pancreatic markers (Pdx1 and Insulin/Insulin-2 mRNA levels) and released C-peptide are near the same or even better than the previous studies. However, the C-peptide amounts released by the pancreatic islets [60] are significantly higher compared to the C-peptide released by our optimal condition, exosome + small molecule combination. The comparison of results in Supplementary Tables S8 and S9 indicate that the differentiation efficiency of current protocol is similar to the protocol for the differentiation from iPSCs, but better than those from MEF (suspension culture). Next, we compared current study with the differentiation study of iPSCs treated with human islet-derived exosome [59]. Interestingly, in the case of exosome treatment on the protocol for the differentiation of iPSC to β-like cells, does not give any significant change in C-peptide production, suggesting islet-derived exosome might contain less amount of key components responsible for β-cell differentiation. Overall, more optimization is required to generate β-like cells using exosome + small molecule or miRNA + small molecule combination that can release C-peptide at par with the human islets.

Our study has some limitations as we did not test the functionality of the differentiated β-like cells in vivo. Accordingly, transplantation of the differentiated β-like cells into model animals of type 1 or 2 diabetes and following observation of glucose level are required for assessment of the clinical application of the current study. In addition, it is revealed that the C-peptide levels released from the MEFs differentiated using exosome + small molecule combination was relatively low compared to the endogenous pancreatic β-cells, which hinders the clinical application of the β-like cells obtained in this study. However, since we have identified the key miRNAs that play a key role in the β-cell differentiation, we believe that further effort will eventually result in the production of the β-like cells with high functionality.

The use of the small molecule-based differentiation approach has become popular in the field of regenerative medicine. By amalgamating the differentiation efficiency of small molecules and exosomes, we developed a new differentiation protocol with enhanced differentiation ability. To our knowledge, this is the first study that directed the differentiation potential of the exosomes for PEC generation from the MEFs, and exocrine-cell differentiation, that was augmented by the inclusion of specific small molecules, to form functional β-like cells. After differentiation into functional pancreatic β-like cells through the use of β-cell-derived exosomes, mouse-derived fibroblasts and exocrine cells could be transplanted in vivo as surrogate β-cells to restore euglycemia in diabetic animal models. Moreover, the delivery of β-cell exosomes into the exocrine compartment could induce the neogenesis of β-like cells. Therefore, β-cell specific, exosome-mediated, cellular differentiation could be used to differentiate various human cell sources into glucose-responsive β-like cells that can alleviate hyperglycemic conditions upon transplantation into diabetic patients.

## Supplementary Materials

The following are available online at https://www.mdpi.com/2227-9059/8/11/485/s1.

## Patents

The results of this study have been filed, Korean Patent 10-2019-0053439.
