# Supplementary material for: Exosome-Mediated Differentiation of Mouse Embryonic Fibroblasts and Exocrine Cells into β-Like Cells and the Identification of Key miRNAs for Differentiation
Source: Biomedicines. 2020 Nov 9;8(11):485. doi: 10.3390/biomedicines8110485 (PMC7695333; doi:10.3390/biomedicines8110485)
Supplement: Supplementary file 1 [file biomedicines-08-00485-s001.pdf]

Supplementary Information

Exosome-mediated differentiation of mouse embryonic fibroblasts and exocrine cells into β-like cells and the identification of key miRNAs for differentiation

Paulami Mandal[1], Debojyoti De[2], Im Dong Uk[3], Sung Hee Um[3], and Kyeong Kyu Kim[1*]

[1]Department of Precision Medicine, Sungkyunkwan University School of Medicine, Suwon 16419, Korea; mandalpaulami28@gmail.com

[2]Department of Biotechnology, National Institute of Technology Durgapur, 713209, India; debojyoti.de@bt.nitdgp.ac.in

[3]Department of Molecular Cell Biology, Sungkyunkwan University School of Medicine, Suwon 16419, Korea; Ldw4545@gmail.com, shum@skku.edu

***Corresponding author: Kyeong Kyu Kim**

email: kyeongkyu@skku.edu; Tel.: (+82) 31 299 6136

Supplementary Tables

Supplementary Table 1: List of primers used in qRT-PCR analysis.

Gene Name	Forward Primer (5′-3′)	Reverse Primer (5′-3′)
Ngn3	CAGTCACCCACTTCTGCTTC	GAGTCGGGAGAACTAGGATG
Nkx6.1	CTTCTGGCCCGGAGTGATG	GGGTCTGGTGTGTTTTCTCTTC
Pdx1	CTTAACCTAGGCGTCGCACAA	GAAGCTCAGGGCTGTTTTTCC
Insulin-1	GACCAGCTATAATCAGAGACCATC	GTAGGAAGTGCACCAACAGG
Insulin-2	GGCTTCTTCTACACACCCAT	CCAAGGTCTGAAGGTCACCT
Glucagon	AGGGACCTTTACCAGTGATGT	AATGGCGACTTCTTCTGGGAA
Elastase1	CGTGGTTGCAGGCTATGACAT	TTGTTAGCCAGGATGGTT
Cytokeratin 19	CCTCCCGAGATTACAACCACT	GGCGAGCATTGTCAATCTGT
β-actin	GGCACCACACCTTCTACAATG	CCATGCCTGTGATTTGCAGTA

Supplementary Table 2. List of the antibodies used in the study.

Antibody	Dilution used	Company	Catalog number	Purpose
Pdx1	1:100	R&D systems	AF2419	IF
Ngn3	1:100	Millipore	AB5684	IF
C-peptide	1:200	Cell Signaling Technology	4593S	IF
β-actin	1:1000	Cell Signaling Technology	4967S	WB
CD9	1:1000	Abcam	ab92726	WB
TSG101	1:1000	Abcam	ab83	WB
Alix	1:1000	Abcam	ab117600	WB
Calnexin	1:1000	Abcam	ab22595	WB

Supplementary Table 3: Comparison of pancreatic markers (Pdx1 and Insulin-2) and MEF marker (Fsp1) expression (qRT-based) during the various differentiation stages in the tested conditions

Marker	Condition	**Stage 1 (MEF to PEC)** (expression fold change)	**Stage 2 (PEC to PPLC)** (expression fold change)	**Stage 3 (PPLC to BLC)** (expression fold change)
**Pdx1** (upregulation compared to starting cells)	Small molecule	2.0	1.9	2.3
	Exosome	3.9	3.9	4.1
	Exosome+small molecule	5.9	6.4	7.1
	*miRNA+small molecule	5.2	4.8	5.6
**Fsp1** (downregulation compared to starting cells)	Small molecule	0.7	0.7	0.6
	Exosome	0.5	0.45	0.35
	Exosome+small molecule	0.45	0.3	0.38
	*miRNA+small molecule	Not detected	Not detected	Not detected
**Insulin-2** (upregulation compared to starting cells)	Small molecule	Not detected	Not detected	3.6
	Exosome	Not detected	Not detected	5.2
	Exosome+small molecule	Not detected	Not detected	10.0
	*miRNA+small molecule	Not detected	Not detected	8.4

***miRNA = miR-127+709 combination 3**

Supplementary Table 4. List of miRNAs with their respective fold changes (MIN6 derived exosome versus MIN6 cells) identified during our miRNA-seq study.

Mature ID	Exosome/Cell.fc
mmu-let-7a-1-3p	-3.807821
mmu-let-7a-5p	-9.116792
mmu-let-7b-3p	-10.570485
mmu-let-7b-5p	-3.527494
mmu-let-7c-2-3p	-3.807912
mmu-let-7c-5p	-6.228514
mmu-let-7d-3p	-17.191155
mmu-let-7d-5p	-14.472401
mmu-let-7e-5p	-8.589144
mmu-let-7f-5p	-15.456174
mmu-let-7g-5p	-17.265257

mmu-let-7i-5p	-12.551729
mmu-miR-106b-3p	-8.050482
mmu-miR-10b-5p	40.133529
mmu-miR-1187	166.915824
mmu-miR-1192	19.580919
mmu-miR-1195	259.496784
mmu-miR-122-5p	80.326061
mmu-miR-125a-5p	-13.613864
mmu-miR-125b-5p	-3.713809
mmu-miR-126a-3p	10.446557
mmu-miR-126a-5p	67.819520
mmu-miR-127-3p	9.042725
mmu-miR-127-5p	104.129920
mmu-miR-128-3p	-16.518765
mmu-miR-129-1-3p	-10.913033
mmu-miR-129-2-3p	-26.433096
mmu-miR-129-5p	-5.983911
mmu-miR-1306-5p	-4.406894
mmu-miR-130a-3p	-4.330568
mmu-miR-130b-3p	-3.431735
mmu-miR-130b-5p	-22.697251
mmu-miR-134-5p	24.086596
mmu-miR-139-5p	14.659340
mmu-miR-143-3p	21.302332
mmu-miR-145a-5p	198.195520
mmu-miR-146b-5p	-4.292185
mmu-miR-149-5p	-5.878755
mmu-miR-150-5p	168.679944
mmu-miR-15b-3p	-24.006716
mmu-miR-15b-5p	-8.999508
mmu-miR-183-5p	-17.122902
mmu-miR-1839-3p	-4.184237
mmu-miR-1839-5p	-7.860892
mmu-miR-186-5p	-3.015661
mmu-miR-187-3p	-7.720757
mmu-miR-1895	77.539874
mmu-miR-1934-3p	26.738363
mmu-miR-1943-5p	10.223467
mmu-miR-1946a	7.675338
mmu-miR-1946b	9.877801
mmu-miR-1983	-10.353028
mmu-miR-199a-3p	60.663636
mmu-miR-199b-3p	60.654103
mmu-miR-19b-3p	178.961694

mmu-miR-200b-3p	-37.577451
mmu-miR-200b-5p	-8.379391
mmu-miR-200c-3p	-25.659959
mmu-miR-21a-5p	-5.893460
mmu-miR-23a-3p	-3.245082
mmu-miR-23a-5p	3.720200
mmu-miR-23b-3p	-18.144918
mmu-miR-25-3p	-12.006030
mmu-miR-26a-5p	-4.261298
mmu-miR-26b-5p	-8.404236
mmu-miR-28a-3p	-16.334221
mmu-miR-28a-5p	-10.068425
mmu-miR-28c	-10.074902
mmu-miR-29a-3p	-12.915652
mmu-miR-29b-3p	-13.628933
mmu-miR-29c-3p	-23.708318
mmu-miR-300-3p	4.861530
mmu-miR-3068-3p	11.984002
mmu-miR-3082-5p	45.686787
mmu-miR-30a-3p	-14.142488
mmu-miR-30c-2-3p	-5.713365
mmu-miR-30c-5p	-11.296544
mmu-miR-30d-5p	-5.357540
mmu-miR-30e-3p	-17.931555
mmu-miR-3102-3p	-20.683334
mmu-miR-3102-3p.23p	-6.690369
mmu-miR-32-3p	-17.335852
mmu-miR-320-3p	3.991324
mmu-miR-323-3p	9.155810
mmu-miR-324-3p	-3.296454
mmu-miR-324-5p	-5.000177
mmu-miR-328-3p	-7.100876
mmu-miR-328-5p	25.980339
mmu-miR-337-5p	5.995661
mmu-miR-339-5p	6.252500
mmu-miR-340-5p	-23.396447
mmu-miR-341-3p	42.271474
mmu-miR-342-3p	-4.876783
mmu-miR-344d-3p	-9.641260
mmu-miR-344g-3p	6.952215
mmu-miR-345-3p	-4.277142
mmu-miR-345-5p	-28.282165
mmu-miR-3470a	355.374470
mmu-miR-3470b	547.665913

mmu-miR-3473a	256.265604
mmu-miR-3473b	92.866008
mmu-miR-3473d	3.092976
mmu-miR-3473e	54.761252
mmu-miR-3473f	37.385475
mmu-miR-361-3p	-13.371978
mmu-miR-374b-5p	-26.222869
mmu-miR-374c-5p	-26.507382
mmu-miR-375-3p	-3.731548
mmu-miR-375-5p	-9.721454
mmu-miR-376b-3p	8.313532
mmu-miR-378a-3p	6.255373
mmu-miR-378a-5p	-6.667246
mmu-miR-378b	4.429897
mmu-miR-378c	3.901886
mmu-miR-378d	3.823223
mmu-miR-379-5p	4.565606
mmu-miR-381-3p	23.917825
mmu-miR-382-3p	11.887733
mmu-miR-382-5p	6.576152
mmu-miR-384-3p	-48.917342
mmu-miR-384-5p	-28.401115
mmu-miR-3960	9.506589
mmu-miR-3963	23.214709
mmu-miR-3968	7.335193
mmu-miR-409-3p	8.735220
mmu-miR-411-3p	3.992497
mmu-miR-421-3p	-7.590791
mmu-miR-423-3p	-4.619359
mmu-miR-423-5p	3.789556
mmu-miR-425-3p	-4.443715
mmu-miR-429-3p	-10.439213
mmu-miR-434-3p	33.493020
mmu-miR-455-3p	-14.756366
mmu-miR-465a-3p	6.568159
mmu-miR-465a-5p	13.014475
mmu-miR-465b-3p	6.568175
mmu-miR-465c-3p	6.567922
mmu-miR-466c-5p	9.622100
mmu-miR-466f-3p	28.142251
mmu-miR-466g	27.847961
mmu-miR-466h-3p	36.489047
mmu-miR-466i-3p	25.019113
mmu-miR-466i-5p	333.000653

mmu-miR-466m-3p	28.729906
mmu-miR-467b-3p	51.993403
mmu-miR-467f	17.702357
mmu-miR-470-5p	38.575463
mmu-miR-483-3p	-18.550653
mmu-miR-483-5p	3.715988
mmu-miR-485-3p	4.261536
mmu-miR-485-5p	22.053647
mmu-miR-486a-5p	616.349384
mmu-miR-486b-5p	616.499206
mmu-miR-494-3p	33.379355
mmu-miR-495-3p	9.182152
mmu-miR-501-3p	-5.544372
mmu-miR-5100	17.896849
mmu-miR-5114	36.382429
mmu-miR-5128	33.905757
mmu-miR-532-3p	-4.630297
mmu-miR-532-5p	-6.477315
mmu-miR-540-3p	11.424586
mmu-miR-541-5p	4.936353
mmu-miR-5615-3p	33.862266
mmu-miR-574-5p	4.578137
mmu-miR-664-3p	-5.381095
mmu-miR-667-3p	25.139104
mmu-miR-669c-3p	59.973546
mmu-miR-669f-3p	10.129424
mmu-miR-669h-3p	18.611816
mmu-miR-669p-3p	11.452058
mmu-miR-671-5p	21.794305
mmu-miR-673-5p	3.753058
mmu-miR-674-5p	-4.451334
mmu-miR-676-3p	-11.571867
mmu-miR-6769b-5p	9.313431
mmu-miR-700-3p	-4.115564
mmu-miR-7033-5p	5.878816
mmu-miR-706	92.986520
mmu-miR-7070-5p	20.795715
mmu-miR-708-5p	-5.330836
mmu-miR-709	97.283987
mmu-miR-741-3p	8.882910
mmu-miR-743a-3p	4.095869
mmu-miR-744-5p	-19.517893
mmu-miR-7651-5p	49.183475
mmu-miR-7667-5p	124.614839

mmu-miR-7668-3p	96.455228
mmu-miR-770-3p	84.832541
mmu-miR-7a-1-3p	-10.662543
mmu-miR-7a-2-3p	-10.126892
mmu-miR-7a-5p	-20.554652
mmu-miR-7b-5p	-17.306686
mmu-miR-8094	91.196484
mmu-miR-8101	12.250054
mmu-miR-871-3p	4.678592
mmu-miR-877-5p	3.306036
mmu-miR-92a-3p	46.780288
mmu-miR-93-3p	-3.244881
mmu-miR-93-5p	-4.742321
mmu-miR-98-3p	-29.624332
mmu-miR-98-5p	-23.672377
mmu-miR-99b-3p	-3.299768
mmu-miR-99b-5p	-18.799197

Supplementary Table 5. Top 20 upregulated miRNAs present in MIN6-derived exosomes and MIN6 parental cells.

**MIN6-derived exosomes**		**MIN6-cells**	
**miRNA**	**Fold-change**	**miRNA**	**Fold change**
mmu-miR-486b-5p	616.499206	mmu-miR-384-3p	48.917342
mmu-miR-486a-5p	616.349384	mmu-miR-200b-3p	37.577451
mmu-miR-3470b	547.665913	mmu-miR-98-3p	29.624332
mmu-miR-3470a	355.374470	mmu-miR-384-5p	28.401115
mmu-miR-466i-5p	333.000653	mmu-miR-345-5p	28.282165
mmu-miR-1195	259.496784	mmu-miR-374c-5p	26.507382
mmu-miR-3473a	256.265604	mmu-miR-129-2-3p	26.433096
mmu-miR-145a-5p	198.195520	mmu-miR-374b-5p	26.222869
mmu-miR-19b-3p	178.961694	mmu-miR-200c-3p	25.659959
mmu-miR-150-5p	168.679944	mmu-miR-15b-3p	24.006716
mmu-miR-1187	166.915824	mmu-miR-29c-3p	23.708318
mmu-miR-7667-5p	124.614839	mmu-miR-98-5p	23.672377
mmu-miR-127-5p	104.129920	mmu-miR-340-5p	23.396447
mmu-miR-709	97.283987	mmu-miR-130b-5p	22.697251
mmu-miR-7668-3p	96.455228	mmu-miR-3102-3p	20.683334
mmu-miR-706	92.986520	mmu-miR-7a-5p	20.554652
mmu-miR-3473b	92.866008	mmu-miR-744-5p	19.517893
mmu-miR-8094	91.196484	mmu-miR-99b-5p	18.799197
mmu-miR-770-3p	84.832541	mmu-miR-483-3p	18.550653
mmu-miR-122-5p	80.326061	mmu-miR-23b-3p	18.144918

Note: Yellow highlighted miRNAs were used for validation experiments.

Supplementary Table 6. miRNAs with almost equal representation in MIN6-derived exosomes and MIN6 parental cells.

Mature ID	Exosome/Cell.fc
mmu-miR-138-2-3p	1.064939
mmu-miR-30e-5p	-1.074367
mmu-miR-3474	1.145588
mmu-miR-411-5p	1.124023
mmu-miR-669o-3p	1.088571
mmu-miR-669a-3p	1.088504
mmu-miR-185-5p	1.009138
mmu-miR-3102-5p.2-5p	1.032621
mmu-miR-1224-3p	-1.034021
mmu-miR-344-3p	1.040514
mmu-miR-690	1.046224
mmu-miR-140-3p	-1.067195
mmu-miR-8114	-1.149219
mmu-miR-505-3p	-1.242743
mmu-miR-187-5p	1.321062
mmu-miR-484	1.122070
mmu-miR-3057-5p	1.147253
mmu-miR-466q	1.140360
mmu-miR-212-3p	1.147324
mmu-miR-210-5p	1.246240
mmu-miR-138-5p	-1.411990
mmu-miR-188-5p	-1.456892
mmu-miR-107-3p	-1.234204
mmu-miR-30a-5p	1.248001
mmu-miR-128-1-5p	1.444100
mmu-miR-124-3p	1.245127
mmu-miR-219a-1-3p	1.412549
mmu-miR-346-5p	1.312924
mmu-miR-25-5p	-1.291698
mmu-miR-3064-5p	1.598874
mmu-miR-3099-3p	1.295892
mmu-miR-500-3p	-1.413544
mmu-miR-760-3p	1.351941
mmu-miR-92b-3p	-1.416943
mmu-miR-5113	1.421578
mmu-miR-5121	1.807264
mmu-miR-6916-5p	-1.960317
mmu-miR-34a-5p	-1.840036

Supplementary Table 7: Marker expression (Pdx1) between the MIN6-derived exosome treated and miRNA combination transfected MEFs.

Marker	Condition	**Expression level** (fold-change compared to medium control cells)	**Expression level** (fold-change compared to miR-127+709+19b combination 1-transfected cells)
**Pdx1**	Exosome-100 µg	4.9	2.0
	miRNA (mir-127+709)-200 nM (combination 3)	3.7	1.6
	miRNA (mir-127+709+19b)-200 nM (combination 1)	2.4	1.0

Supplementary Table 8. Comparison of Pdx1 and Insulin markers in different studies compared to our study.

**Conditions**	**Current study**	**Current study**	**Kim *et al.* study [1]**	**Chandravanshi *et al.* study [2]**
**Starting cells**	MEFs	MEFs	MEF (NOD mice)	MEFs
**Method**	Small molecule+ exosome; monolayer culture	Small molecule+ miRNA; monolayer culture	iPSC+small molecule; suspension culture	Small molecule; suspension culture
**Markers**	**Fold change (expression in the final stage)**			
**Pdx1** (mRNA)	8.1	5.6	10.0	5.5
**Insulin-1+2** (mRNA)	11.7	8.4	6.0	8.0*

***Insulin-2 only**

Supplementary Table 9: Comparison of C-peptide release in different studies compared to our study.

	**Our study**	**Chandra *et al.* study [3]**	**Zhu *et al.* study [4]**	**Lima *et al.* study [5]**
**Starting cells**	MEFs	Human adipose derived stem cells (hADSC)	Human fibroblasts	Human islets (1500 islet equivalents)
**Method used**	Exosome+small molecule; monolayer culture	Small molecule; suspension culture	Small molecule; transient iPSC; monolayer + suspension culture	Transplanted in the kidney capsule of SCID mice
**C-peptide release** (absolute amount)	145.4 pM (low glucose)<br><br>180 pM (high glucose)	47.68 pM (low glucose)<br><br>80.61 pM (high glucose)	60 pM (low glucose)<br><br>90 pM (high glucose)	1000 pM (0 minutes after intraperitoneal glucose tolerance test; low glucose<br><br>1500 pM (60 minutes after intraperitoneal glucose tolerance test; high glucose

Supplementary Figures

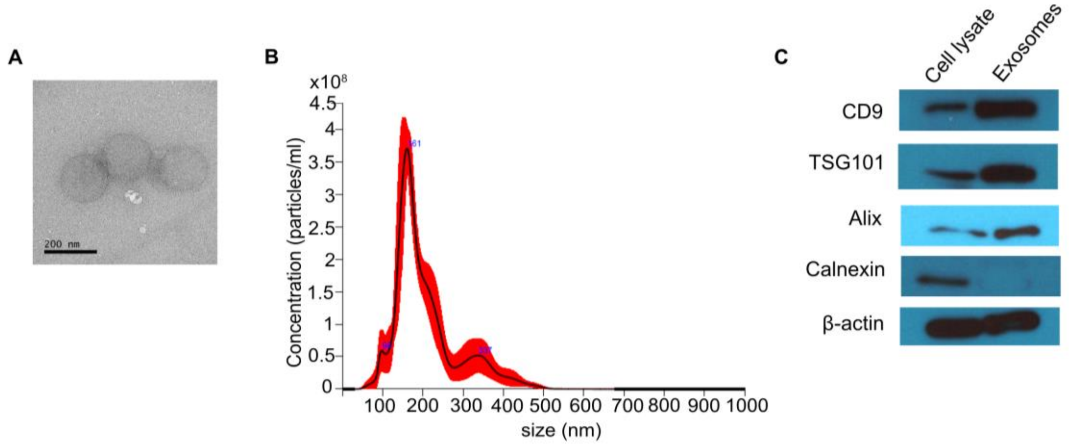

Supplementary Figure 1. Characterization of exosomes derived from the conditioned medium of the MIN6 insulinoma cell line. (**A**) Morphology and size of MIN6-derived exosomes as determined by TEM negative staining. Scale bar = 200 nm. (**B**) Particle size distribution of the MIN6-derived exosomes using NTA. (**C**) Western blot analysis of exosome-associated proteins—CD63, CD9, TSG101, and Alix. Calnexin is an endoplasmic reticulum protein. β-actin was used as a loading control. The data shown represent three (A and B), and two (C) independent experiments. TEM = transmission electron microscopy, NTA = nanoparticle tracking analysis. The previous western blot image for ALIX has been replaced with a new image.

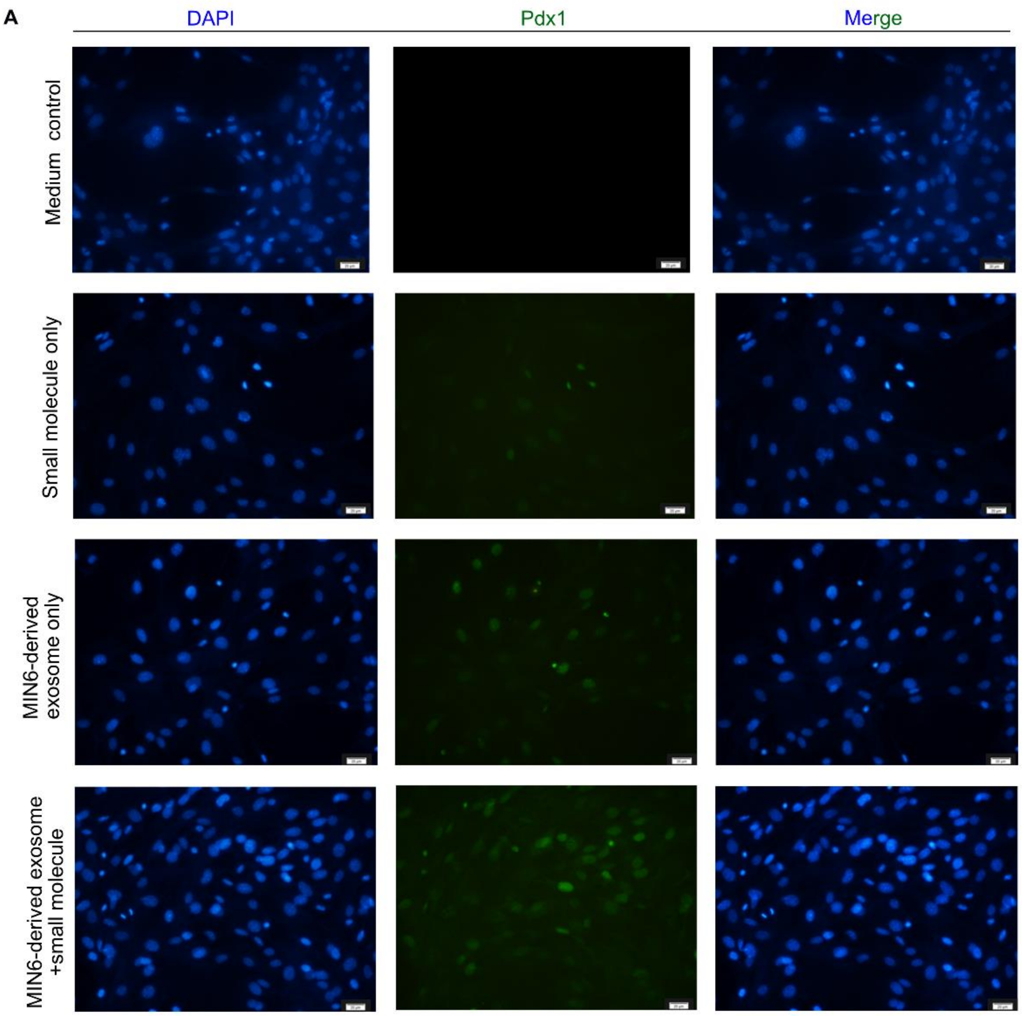
A
DAPI
Pdx1
Merge
Medium control
Small molecule only
MIN6-derived exosome only
MIN6-derived exosome +small molecule

B

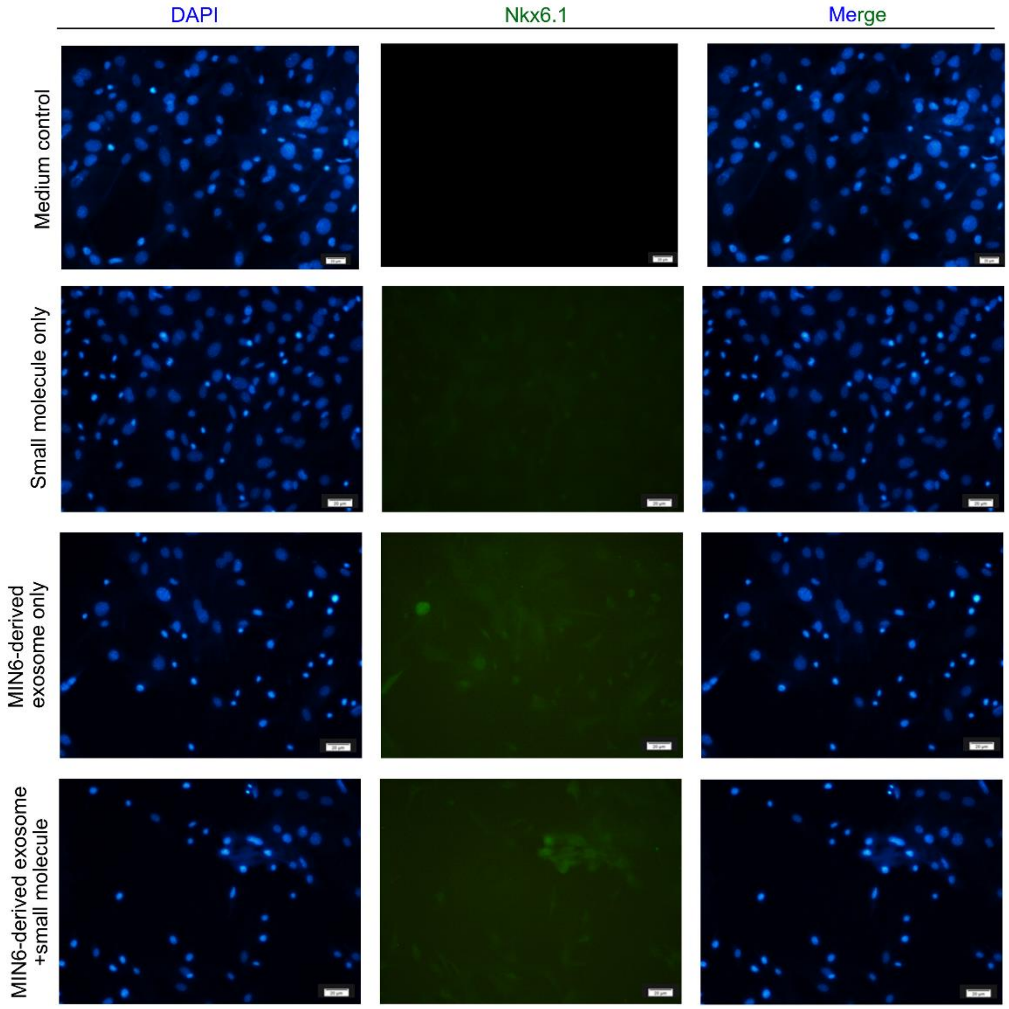
DAPI
Nkx6.1
Merge
Medium control
Small molecule only
MIN6-derived
exosome only
MIN6-derived exosome
+small molecule

C

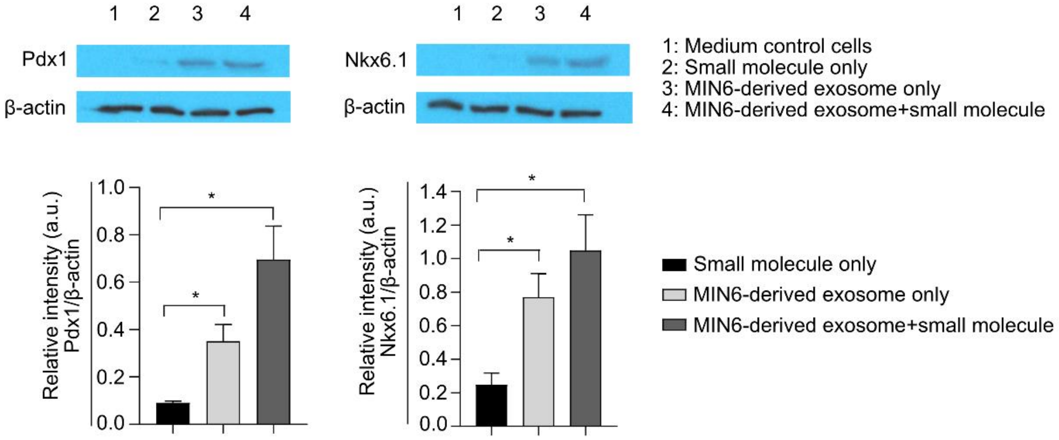

D

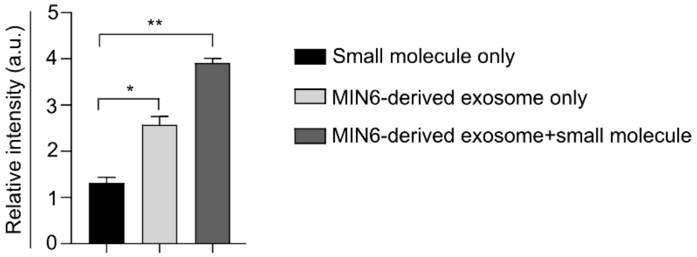

Supplementary Figure 2. Expression of the Pdx1 and Nkx6.1 protein in MEFs differentiated into β-like cells. (**A**) Pdx1 protein expression in MEFs differentiated into β-like cells under various conditions. Scale bar = 20 μm. (**B**) Nkx6.1 protein level in MEFs differentiated into β-like cells. Scale bar = 20 μm. (**C**) Western blot images for Pdx1 and Nkx6.1 protein present in cells grown under different conditions (Top). Densitometry analysis was performed to quantify the protein levels in different conditions (Bottom). As no Pdx1 and Nkx6.1 band was detected in medium control cells, they were not considered for the densitometry analysis. (**D**) Fluorescence intensity was measured using ImageJ software for the C-peptide positive cells grown under different conditions relative to medium control cells. a.u.= arbitrary units. $*P < 0.05$, and $**P < 0.01$. Statistical significance was determined by paired, two-tailed t-testing. The data shown represent the mean ± SEM of two independent experiments.

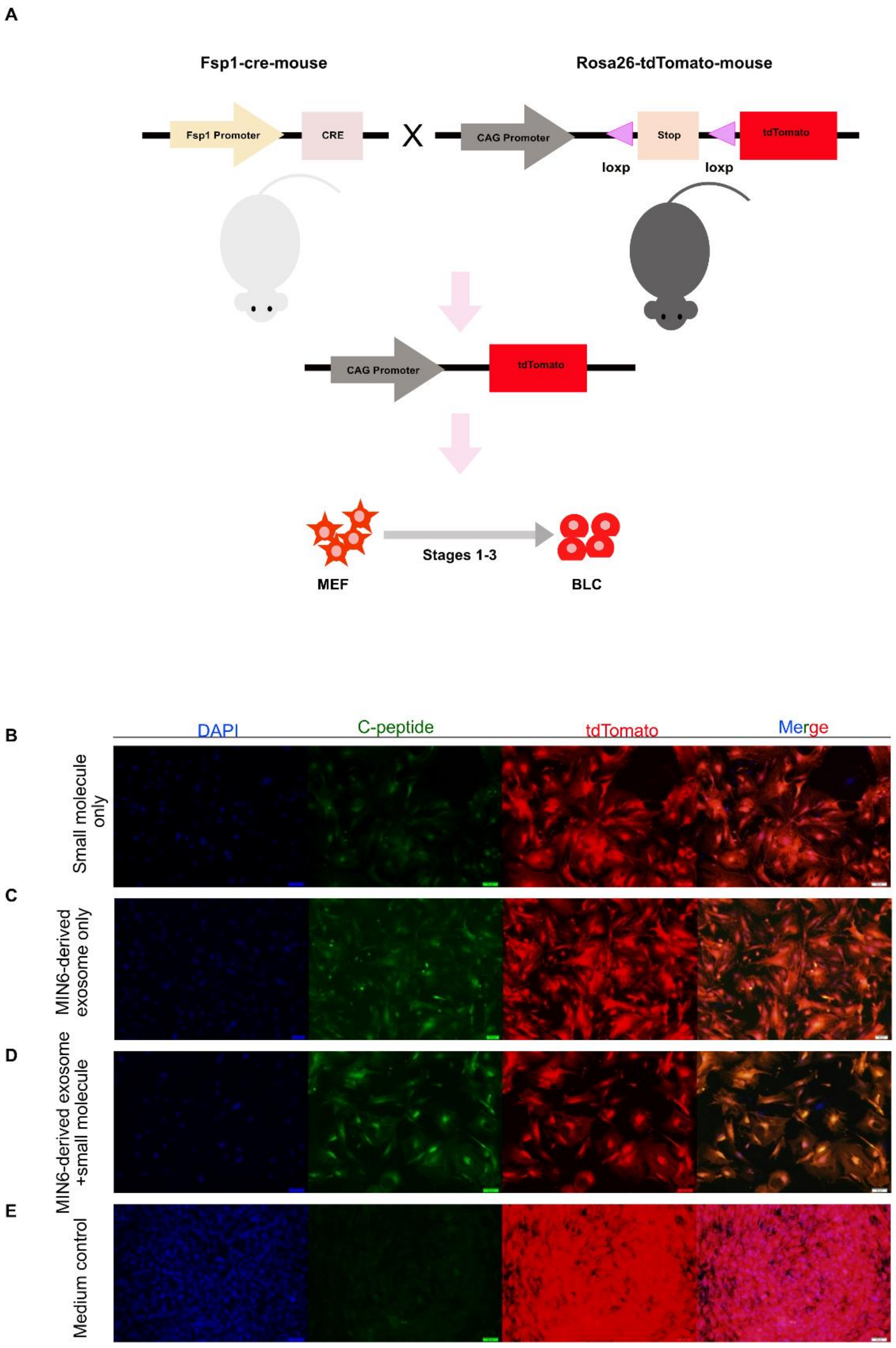
A
Fsp1-cre-mouse
Rosa26-tdTomato-mouse
Fsp1 Promoter
CRE
X
CAG Promoter
Stop
tdTomato
loxp
loxp
CAG Promoter
tdTomato
Stages 1-3
MEF
BLC
B
C
D
E
DAPI
C-peptide
tdTomato
Merge
Small molecule only
MIN6-derived exosome only
MIN6-derived exosome +small molecule
Medium control

Supplementary Figure 3. tdTomato MEFs undergo differentiation to β-like cells. (**A**) Schematic diagram depicting the lineage tracing experiment. The tdTomato MEFs were differentiated into β-like cells using (**B**) small molecule only, (**C**) MIN6-derived exosome only, and (**D**) MIN6-derived exosome + small molecule methods. (**E**) Medium control cells. Scale bar = 50 μm. The first panel represents DAPI (nucleus) stained cells; the second panel represents C-peptide stained cells; the third panel depicts tdTomato positive cells, and the last panel shows the co-expression of C-peptide with tdTomato (merged image), indicating the fibroblastic origin of the differentiated β-like cells. The data shown represent two independent experiments. MEF = mouse embryonic fibroblast, and BLC= β-like cells.

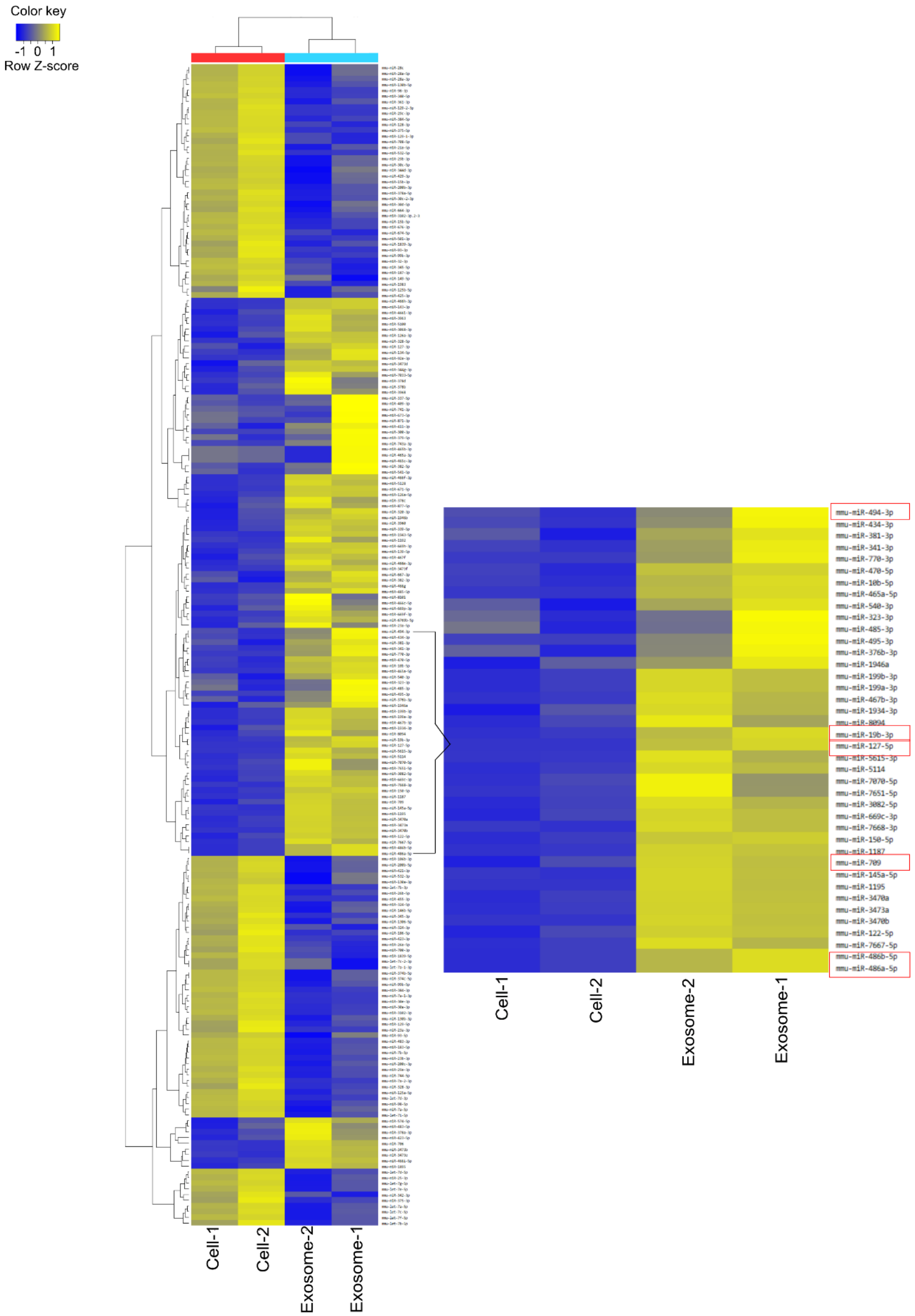
Color key
-1 0 1
Row Z-score
mmu-miR-494-3p
mmu-miR-434-3p
mmu-miR-381-3p
mmu-miR-341-3p
mmu-miR-770-3p
mmu-miR-470-5p
mmu-miR-10b-5p
mmu-miR-465a-5p
mmu-miR-540-3p
mmu-miR-323-3p
mmu-miR-485-3p
mmu-miR-495-3p
mmu-miR-376b-3p
mmu-miR-1946a
mmu-miR-199b-3p
mmu-miR-199a-3p
mmu-miR-467b-3p
mmu-miR-1934-3p
mmu-miR-8094
mmu-miR-19b-3p
mmu-miR-127-5p
mmu-miR-5615-3p
mmu-miR-5114
mmu-miR-7070-5p
mmu-miR-7651-5p
mmu-miR-3082-5p
mmu-miR-669c-3p
mmu-miR-7668-3p
mmu-miR-150-5p
mmu-miR-1187
mmu-miR-709
mmu-miR-145a-5p
mmu-miR-1195
mmu-miR-3470a
mmu-miR-3473a
mmu-miR-3470b
mmu-miR-122-5p
mmu-miR-7667-5p
mmu-miR-486b-5p
mmu-miR-486a-5p
Cell-1
Cell-2
Exosome-2
Exosome-1

Supplementary Figure 4. Heatmap of differential miRNA expression profiles. The miRNAs derived from miRNA-sequencing of MIN6 cells and their exosomes were clustered using one-way hierarchical clustering (Euclidean method, complete Linkage) and the Z-score for the normalized value (log_2 based). Yellow indicates upregulation, and blue represents downregulation of miRNA expression. The region representing the miRNAs subsequently validated in our study has been enlarged, and the miRNAs are marked with red boxes.

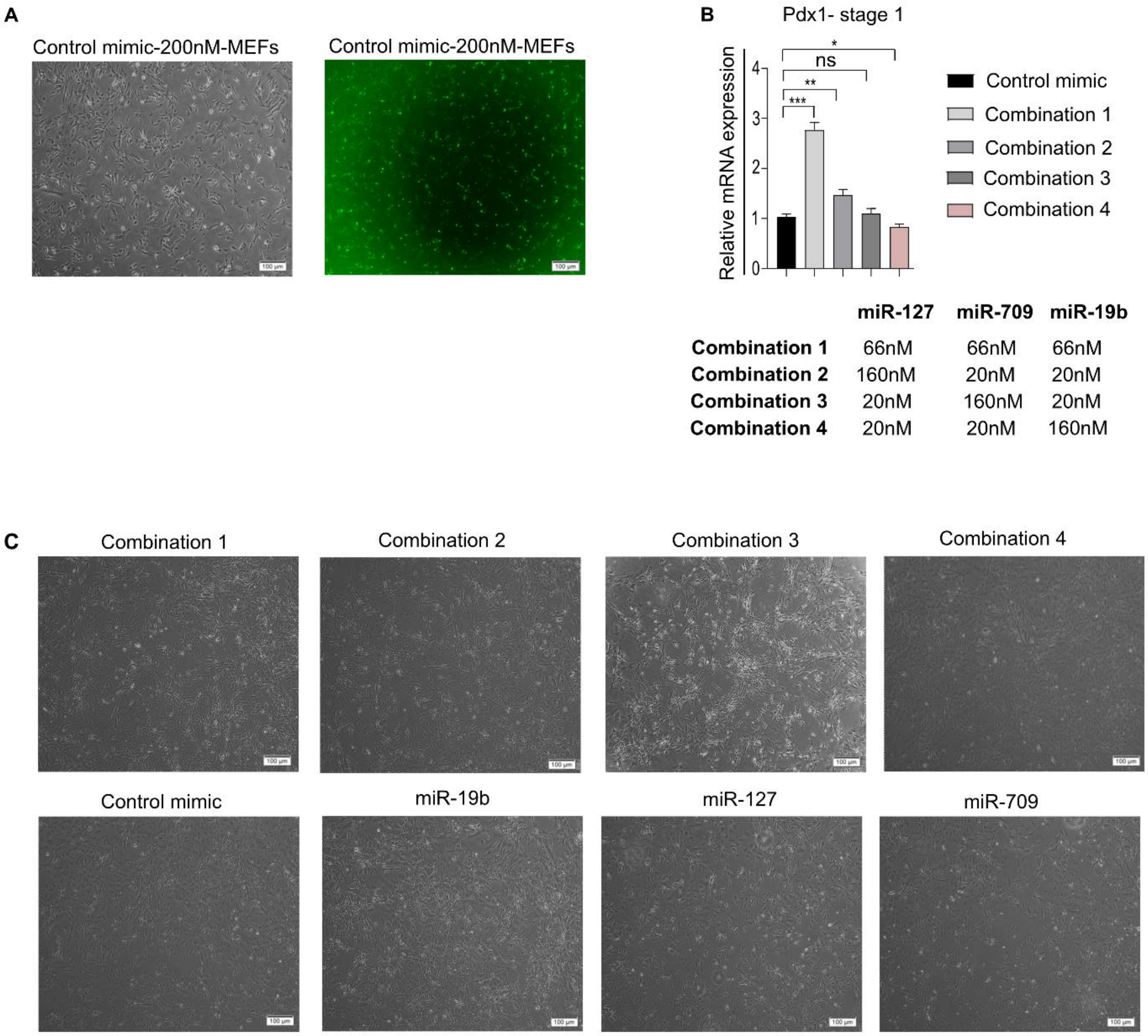

Supplementary Figure 5. Transfection of miRNA mimics and their combinations. (**A**) Transfection of 5′ FAM labeled control mimic to optimize transfection efficiency in MEFs; the first panel is a bright field image of the MEFs 48 hours after transfection in stage 1 medium, and the second panel is the corresponding green channel (fluorescence microscope) image. Scale bar = 100 μm. (**B**) Pdx1 transcript induction using different combinations of miR-127, miR-709, and miR-19b in stage 1 medium, as determined by a qRT-PCR analysis (upper panel). $*P < 0.05$, $**P < 0.01$, and $***P < 0.001$. Statistical significance was determined by paired, two-tailed t-testing. The data shown represent the mean ± SEM of three independent experiments. (**C**) Bright field microscopic images depicting morphological changes in MEFs upon treatment with different combinations (upper panel) in stage 1 of differentiation (after 48 hours). The combinations were made using different ratios of the three miRNAs. Individual transfections (lower panel) of miR-127, miR-709, and miR-19b were used as references to assess the proliferative ability of stage 1 MEFs treated with various combinations. Scale bar = 100 μm. ns = non-significant.

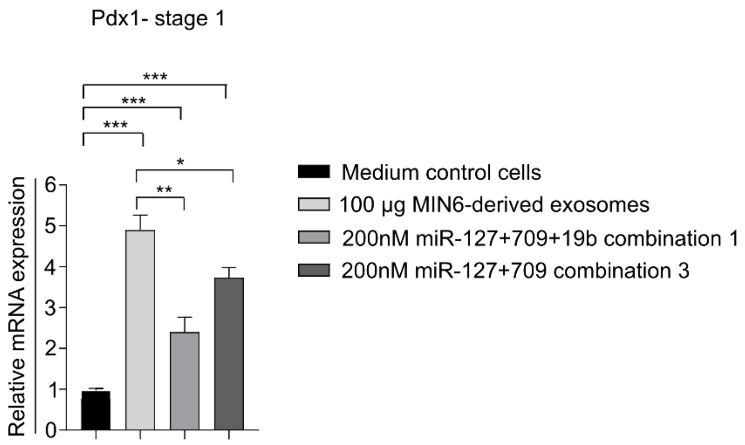

Supplementary Figure 6. Pdx1 expression induced by MIN6-derived exosome and different miRNA combinations. MIN6-derived exosomes (100 μg; saturating exosome amount), miR-127+709+19b combination 1, and miR-127+709 combination 3 were used to treat MEFs in stage 1 for measuring induction of Pdx1 expression compared to medium control cells. $*P < 0.05$, $**P < 0.01$, and $***P < 0.001$. Statistical

significance was determined by paired, two-tailed t-testing. The data shown represent the mean ± SEM of two independent experiments.

Supplemental References:

[1] Jeon, K.; Lim, H.; Kim, J.-H.; Thuan, N.V.; Park, S.H.; Lim, Y.-M.; Choi, H.-Y.; Lee, E.-R.; Kim, J.-H.; Lee, M.-S.; et al. Differentiation and Transplantation of Functional Pancreatic Beta Cells Generated from Induced Pluripotent Stem Cells Derived from a Type 1 Diabetes Mouse Model. Stem Cells and Development 2012, 21, 2642–2655, doi:10.1089/scd.2011.0665

[2] Chandravanshi, B.; Bhonde, R. Reprogramming mouse embryo fibroblasts to functional islets without genetic manipulation. J Cell Physiol 2018, 233, 1627–1637, doi:10.1002/jcp.26068

[3] Chandra, V.; G, S.; Muthyala, S.; Jaiswal, A.K.; Bellare, J.R.; Nair, P.D.; Bhonde, R.R. Islet-Like Cell Aggregates Generated from Human Adipose Tissue Derived Stem Cells Ameliorate Experimental Diabetes in Mice. PLoS ONE 2011, 6, e20615, doi:10.1371/journal.pone.0020615

[4] Zhu, S.; Russ, H.A.; Wang, X.; Zhang, M.; Ma, T.; Xu, T.; Tang, S.; Hebrok, M.; Ding, S. Human pancreatic beta-like cells converted from fibroblasts. Nature Communications 2016, 7, 10080, doi:10.1038/ncomms10080

[5] Lima, M.J.; Docherty, H.M.; Chen, Y.; Vallier, L.; Docherty, K. Pancreatic Transcription Factors Containing Protein Transduction Domains Drive Mouse Embryonic Stem Cells towards Endocrine Pancreas. PLoS ONE 2012, 7, e36481, doi:10.1371/journal.pone.0036481.
